# Supplementary material for: A plug-and-play platform of ratiometric bioluminescent sensors for homogeneous immunoassays
Source: Nat Commun. 2021 Jul 28;12:4586. doi: 10.1038/s41467-021-24874-3 (PMC8319308; doi:10.1038/s41467-021-24874-3)
Supplement: Supplementary file 1 — Supplementary Information [file 41467_2021_24874_MOESM1_ESM.pdf]

Supplementary information

A plug-and-play platform of ratiometric bioluminescent sensors for homogeneous immunoassays

Yan Ni[‡1,2], Bas J.H.M. Rosier[‡1,2], Eva A. van Aalen[‡1,2], Eva T.L. Hanckmann[1,2], Lieuwe Biewenga[1,2], Anna-Maria Makri Pistikou[2,3], Bart Timmermans[1,2], Chris Vu[1,2], Sophie Roos[1,2], Remco Arts[1,2], Wentao Li[4], Tom F.A. de Greef[1,2,3,5], Marcel M.G.J. van Borren[6], Frank J. M. van Kuppeveld[4], Berend-Jan Bosch[4], Maarten Merkx[1,2]*

[1.] Laboratory of Chemical Biology, Department of Biomedical Engineering, Eindhoven University of Technology, The Netherlands.
[2.] Institute for Complex Molecular Systems, Eindhoven University of Technology, The Netherlands.
[3.] Computational Biology Group, Department of Biomedical Engineering, Eindhoven University of Technology, The Netherlands.
[4.] Virology Section, Infectious Diseases and Immunology Division, Department of Biomolecular Health Sciences, Faculty of Veterinary Medicine, Utrecht University, Utrecht, the Netherlands.
[5.] Institute for Molecules and Materials, Radboud University, Heyendaalseweg 135, 6525 AJ Nijmegen, The Netherlands.
[6.] Department of Clinical Chemistry, Rijnstate Hospital, Arnhem, the Netherlands.

‡ These authors contributed equally.
*Correspondence should be addressed to m.merkx@tue.nl.

Table of Contents

Supplementary note 1 4

Protocol for sensor protein production 4

Supplementary note 2 6

Thermodynamic model 6

Supplementary Figure 1. Thermodynamic scheme describing the interactions in the assay mixture. 6

Supplementary Figure 2. Speciation plot illustrating the concentration of various species versus analyte concentration. 8

Supplementary Figure 3. Simulation of response curve for different parameters. 9

Supplementary Figure 4. Experimental data fitting to the model. 10

Supplementary figures 11

Supplementary Figure 5. Schematic representation of protein G-mediated photoconjugation. 11

Supplementary Figure 6. SDS-PAGE analysis of Gx-LB and Gx-SB purification. 11

Supplementary Figure 7. ESI-QTOF mass spectra of Gx-LB and Gx-SB proteins. 12

Supplementary Figure 8. CTnI dose-dependency using different sensor concentrations 13

Supplementary Figure 9. CTnI dose-dependency using Gx-SB variants. 14

Supplementary Figure 10. Time dependence of intensiometric and ratiometric assays for cTnI. 15

Supplementary Figure 11. Ratiometic assays of cTnI using different concentrations of calibrator luciferase. 16

Supplementary Figure 12. Kinetics of intensiometric and ratiometric detection in a one-step assay for cTnI. 17

Supplementary Figure 13. Intensiometric assays of cTnI in human blood plasma. 18

Supplementary Figure 14. Comparison of cTnI assays in human blood plasma and serum. 18

Supplementary Figure 15. Non-reducing SDS-PAGE analysis of photoconjugation of anti-CRP antibodies with Gx-LB and Gx-SB. 19

Supplementary Figure 16. Kinetics of intensiometric one-step assay for CRP. 19

Supplementary Figure 17. Effect of temperature on NanoLuc and calibrator luciferase (mNG-NL) activity. 20

Supplementary Figure 18. Effect of temperature on intensiometric and ratiometric sensor output of the CRP assay. 21

Supplementary Figure 19. Calibration of RAPPID and ELISA assays for CRP. 22

Supplementary Figure 20. Quantification of CRP in patient plasma samples. 23

Supplementary Figure 21. Non-reducing SDS-PAGE analysis of therapeutic antibodies photoconjugated with Gx-LB and Gx-SB. 24

Supplementary Figure 22. Kinetics of intensiometric and ratiometric one-step assays for anti-infliximab. 24

Supplementary Figure 23. RAPPID assays for infliximab using anti-infliximab conjugated sensor proteins. 25

Supplementary Figure 25. Kinetics of the intensiometric and ratiometric one-step assays for Adalimumab. 27
Supplementary Figure 26. Kinetics of the intensiometric and ratiometric one-step assays for Infliximab. 28
Supplementary Figure 27. Non-reducing SDS-PAGE analysis of anti-SARS-Cov-2 antibodies photoconjugated with Gx-LB and Gx-SB. 29
Supplementary Figure 28. Non-reducing SDS-PAGE analysis of commercial SARS-Cov-2 antibodies photoconjugated with Gx-LB and Gx-SB. 29
Supplementary Figure 29. Ratiometric assays of spike protein using commercial SARS-COV-2 antibodies conjugated with Gx-SB and Gx-LB. 30
Supplementary Figure 30. SDS-PAGE analysis of RBD-SB and RBD-LB purification. 30
Supplementary Figure 31. Ratiometric sensor response for anti-SARS-CoV-2-spike antibody 49F1. 31
Supplementary Figure 32. Kinetics of one-step intensiometric and ratiometric assays for anti-SARS-CoV-2-spike antibody Sino D001. 31
Supplementary Figure 33. DNA and amino acid sequence of Gx-LB. 32
Supplementary Figure 34. DNA and amino acid sequence of Gx-SB. 32
Supplementary Figure 35. DNA and amino acid sequences of SB in Gx-SB variants. 33
Supplementary Figure 36. DNA and amino acid sequence of TNFα-SB. 33
Supplementary Figure 37. DNA and amino acid sequence of RBD-LB.. 34

Supplementary tables 36

Supplementary Table 1. Sequences of primers used. 36
Supplementary Table 2. Overview of RAPPID assays for various protein targets. 37
Supplementary Table 3. Quantification of CRP in 40 patient serum samples by using RAPPID and clinical method. 39

Supplementary references 40

Supplementary note 1

Protocol for sensor protein production

Expression and purification of protein G fusion proteins

1. The pET28a(+) vectors containing DNA encoding for Gx-SB and Gx-LB were ordered from GenScript (see Supplementary Figure 33, 34 and 35 for DNA and amino acid sequences). The pET28a plasmid encoding either Gx-LB or Gx-SB was co-transformed into *E. coli* BL21 (DE3) chemically competent cells (Novagen) together with a pEVOL-pBPA vector encoding a tRNA/tRNA synthetase pair (Addgene plasmid # 31190) according to the manufacturer's instruction.
2. Bacterial cells were initially grown in 50 mL of LB media containing 30 µg/mL kanamycin and 25 µg/mL chloramphenicol, incubated overnight at 250 rpm, 37 ˚C.
3. The overnight cell culture was inoculated into 1 liter 2YT medium (16 g peptone, 5 g NaCl, 10 g yeast extract per liter) containing 30 µg/mL kanamycin and 25 µg/mL chloramphenicol. The culture was incubated at 150 rpm, 37 ˚C until OD_{600} reached 0.3.
4. For para-benzoyl-phenylalanine (pBPA) incorporation, pBPA powder stock (Bachem, F-2800.0001) was added into the culture at a final concentration of 1 mM. The culture was further incubated at 150 rpm, 37 ˚C until OD_{600} reached 0.6.
5. For protein expression, isopropyl β-D-1-thiogalactopyranoside (IPTG) was added into the culture to a final concentration of 0.1 mM and arabinose was added to a final concentration of 0.2% (w/v). The culture was then incubated overnight at 150 rpm, 20 ˚C.
6. Cells were harvested by centrifuging the overnight culture at 10,000 *g* for 10 min.
7. The collected cells were lysed in 10 mL Bugbuster reagent (Novagen) containing 10 µL Benzonase (Novagen) which was gently shaken at room temperature for 30 minutes.
8. Cell lysate was centrifuged at 20,000 *g* for 40 minutes at 4 ˚C and the supernatant was collected.
9. A Ni-NTA column was packed to a bed volume of approximately 5 mL by using Ni-NTA agarose (Qiagen) 50% slurry. The column was equilibrated with 5 column volumes (CVs) of binding buffer (20 mM Tris-HCl, 500 mM NaCl, 5 mM imidazole, pH 8.0) and the collected supernatant was loaded on the column. After washing the column with 5 CVs of wash buffer (20 mM Tris-HCl, 500 mM NaCl, 30 mM imidazole, pH 8.0), the His-tagged fusion protein was eluted from the Ni-NTA column by using 2 CVs of elution buffer (20 mM Tris-HCl, 500 mM NaCl, 500 mM imidazole, pH 8.0).
10. A Strep-Tactin column was packed to a bed volume of approximately 2 mL by using Strep-Tactin®XT Superflow® resin (Iba) 50% slurry. The column was equilibrated with 5 CVs of buffer W (100 mM Tris-HCl, 150 mM NaCl, 1 mM EDTA, pH 8.0) and the collected elution fraction from the Ni-NTA column was loaded on the Strep-Tactin column. After washing the column with 5 CVs of buffer W, the fusion protein was eluted from the column by using 3 CVs of buffer BXT (100 mM Tris-HCl, 150 mM NaCl, 1 mM EDTA, 50 mM Biotin, pH 8.0).
11. The collected elution fraction was concentrated using Amicon® Ultra 15 mL filter with 10 kDa NMWL (Merckmillipore). The protein concentration was determined by measuring absorbance at 280 nm using a NanoDrop 1000 spectrophotometer, and calculated using Beer-Lambert law with the extinction coefficient of 35410 $M^{-1}cm^{-1}$ for Gx-LB and 16960 $M^{-1}cm^{-1}$ for Gx-SB, respectively. The concentrated protein solution was aliquoted, snap-frozen and stored at -80 ˚C until use.
12. The purified proteins were analyzed on SDS-PAGE using Mini-PROTEAN® TGX^{TM} precast gels (4-20%, BioRad). The gels were run in Tris-glycine-SDS running buffer (25 mM Tris, 192 mM glycine, 0.1% w/v SDS, pH 8.3, Bio-Rad) at 140V for 1 hour, and stained using Biosafe Coomassie Brilliant Blue G-250 Gel Staining Solution (Bio-Rad). Subsequently, the SDS-PAGE gel was captured using GE Healthcare ImageQuant Capure v.1.0.2.
13. Correct incorporation of pBPA was confirmed by Q-ToF LC-MS (Supplementary Figure 7) using a High Resolution LC-MS system consisting of a Waters ACQUITY UPLC I-Class system coupled to a Xevo G2 Quadrupole Time of Flight. The proteins were analyzed on a C18A reverse phase column (2.0 x 100mm, Agilent) at 0.3 min/mL by gradient elution using

acetonitrile-water (15:85 to 75:25 v/v) with 0.1% v/v formic acid. Deconvolution of the resulting m/z spectra was performed using MagTran v1.03 software.

Photoconjugation of protein G fusion proteins with antibodies

1. The purified protein G fusion protein (either Gx-LB or Gx-SB) was mixed with IgG antibody in PBS buffer (137 mM NaCl, 2.7 mM KCl, 10 mM Na_2HPO_4, and 1.8 mM KH_2PO_4, pH 7.4) at the molar ratio of 1 IgG to 1~4 Gx-LB/SB in a 0.2 mL PCR tube. The final concentration of IgG in the mixture varied between 0.5 µM to 20 µM, without obvious influence on the photoconjugation efficiency.
2. The mixture was placed on an ice bath and irradiated for 30 to 180 minutes with 365 nm UV light using a Promed UVL-30 UV light source (4×9 watt). The PCR tube was placed close (~1 cm distance) to the UV light. The ice bath was refreshed every 30 minutes to keep the photoconjugation mixture cold.

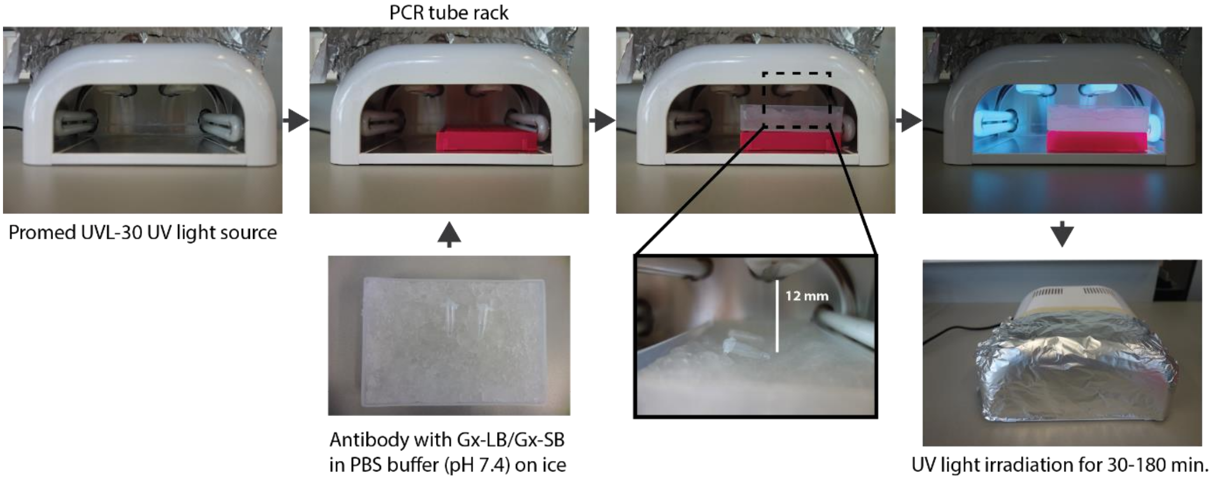

3. If necessary, the photoconjugated products were further purified using 0.2 mL HisPur™ Ni-NTA spin columns (ThermoFisher) according to the manufacturer's instructions. To remove imidazole, the collected elution fraction from Ni-NTA spin columns were desalted on a PD MidiTrap G10 desalting columns (GE Health) equilibrated with PBS buffer (pH 7.4). The collected elution fraction was further concentrated using Amicon® Ultra 0.5 mL filters with 100 kDa NMWL (Merckmillipore). The protein concentration was determined by measuring absorbance at 280 nm and using the extinction coefficient of 245000 $M^{-1}cm^{-1}$ for Ab-LB and 227000 $M^{-1}cm^{-1}$ for Ab-SB, respectively.
4. The antibody-conjugates were analyzed by SDS-PAGE using Mini-PROTEAN® TGX™ precast gels (4-20%, BioRad), and the aliquoted protein solutions were stored at 4 ˚C or -80˚C until use.

Supplementary note 2

Thermodynamic model

To provide a better insight into the response behavior of the RAPPID sensor, we have developed a thermodynamic model that describes the equilibria involved in the analyte-induced heterodimerization of the sensor proteins. We consider a reaction scheme containing three monomeric species: one monovalent protein target (denoted by "T") and two bivalent antibodies which are conjugated with split NanoLuc fragments and contain two identical antigen binding sites (denoted by "A" and "B") (Supplementary Figure 1). The non-conjugated and bi-conjugated antibodies are not considered in the model. As the antibody pair binds to distinct regions of the protein target, we assume that the system is non-cooperative: the split NanoLuc-conjugated antibody (A or B) binds the analyte-bound binary complex (TB or TA) and the free analyte (T) with the same affinity. Following the formation of the analyte-bound ternary complex (TABi), the effective molarity (EM)[1] is introduced to describe the intramolecular association of the split NanoLuc fragments to form the enzymatically active TABa complex. Despite of the possible formation of dual- and triple-analyte-bound quaternary and pentanary complex (T_2ABi and T_3ABi), we assume their concentration is low and thus omit them from the model. The intermolecular interaction of the split NanoLuc leads to formation of a luminescent binary complex (AB) which contributes to the background signal. In experimental assays, the large fragment of split NanoLuc exhibits residual NanoLuc activity, and therefore species containing LB also contribute to this background signal in the absence of analyte.

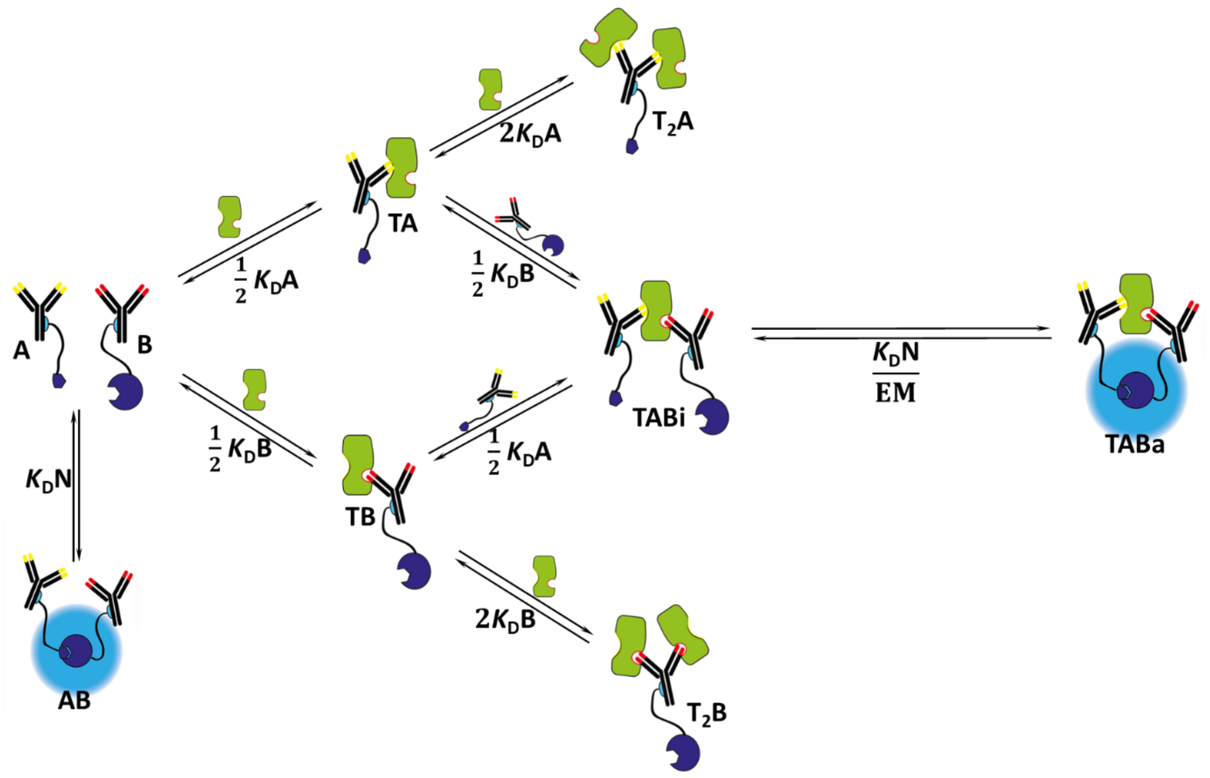

Supplementary Figure 1. Thermodynamic scheme describing the interactions in the assay mixture.

$[A]_0$: total concentration of Gx-SB conjugated antibody

$[B]_0$: total concentration of Gx-LB conjugated antibody

$[T]_0$: total concentration of analyte

$K_{D,A}$: dissociation equilibrium constant for the binding of antibody-SB to analyte

$K_{D,B}$: dissociation equilibrium constant for the binding of antibody-LB to analyte

$K_{D,N}$: dissociation equilibrium constant for the binding of SB to LB fragment

EM: effective molarity

[A]: free concentration of antibody-SB

[B]: free concentration of antibody-LB

[T]: free concentration of analyte

[TA]: free concentration of analyte-bound antibody-SB binary complex

[TB]: free concentration of analyte-bound antibody-LB binary complex

[AB]: free concentration of analyte-unbound sensor protein binary complex

[T_2A]: free concentration of duple-analyte-bound antibody-SB complex

[T_2B]: free concentration of duple-analyte-bound antibody-LB complex

[TABi]: free concentration of ternary complex in inactive state

[TABa]: free concentration of ternary complex in active state

The mass balance of the total concentration of sensor proteins and analyte is expressed in terms of free forms and complexes.

$$[A]_0 = [A] + [AB] + [TA] + [T_2A] + [TABi] + [TABa] \quad \text{(S1)}$$

$$[B]_0 = [B] + [AB] + [TB] + [T_2B] + [TABi] + [TABa] \quad \text{(S2)}$$

$$[T]_0 = [T] + [TA] + [TB] + 2[T_2A] + 2[T_2B] + [TABi] + [TABa] \quad \text{(S3)}$$

Since there are two possible equilibria between a bivalent antibody and monomeric protein target, we derive expressions of the monovalent dissociation constant $K_{D,A}$ and $K_{D,B}$ by taking into account the appropriate statistical factors[1–3]. They can be expressed as follows:

$$\frac{1}{2}K_{D,A} = \frac{[A]*[T]}{[TA]} \quad \text{(S4)}$$

$$2K_{D,A} = \frac{[TA]*[T]}{[T_2A]} \quad \text{(S5)}$$

$$\frac{1}{2}K_{D,B} = \frac{[B]*[T]}{[TB]} \quad \text{(S6)}$$

$$2K_{D,B} = \frac{[TB]*[T]}{[T_2B]} \quad \text{(S7)}$$

$$\frac{1}{2}K_{D,A} = \frac{[A]*[TB]}{[TABi]} \quad \text{(S8)}$$

$$\frac{1}{2}K_{D,B} = \frac{[B]*[TA]}{[TABi]} \quad \text{(S9)}$$

$$K_{D,N} = \frac{[A]*[B]}{[AB]} \quad \text{(S10)}$$

$$K_{D,N} = \frac{[TABi]*EM}{[TABa]} \quad \text{(S11)}$$

One equation among S4-S11 is redundant. When the total concentrations of proteins $[A]_0$, $[B]_0$, $[T]_0$ and dissociation equilibrium constants $K_{D,A}$, $K_{D,B}$, $K_{D,N}$ and effective molarity EM are assigned, there are ten variables: equilibrium concentrations [A], [B], [T], [AB], [TA], [TB], [T_2A], [T_2B], [TABi] and [TABa]. The last seven variables can be expressed in the terms of [A], [B], [T] and dissociation constants.

$$[AB] = \frac{[A]*[B]}{K_{D,N}} \quad \text{(S12)}$$

$$[TA] = \frac{2[A]*[T]}{K_{D,A}} \quad \text{(S13)}$$

$$[TB] = \frac{2[B]*[T]}{K_{D,B}} \quad \text{(S14)}$$

$$[T_2A] = \frac{[A]*[T]^2}{(K_{D,A})^2} \quad \text{(S15)}$$

$$[T_2B] = \frac{[B]*[T]^2}{(K_{D,B})^2} \quad \text{(S16)}$$

$$[TABi] = \frac{4[A]*[B]*[T]}{K_{D,A}*K_{D,B}} \quad \text{(S17)}$$

$$[TABa] = \frac{4[A]*[B]*[T]*EM}{K_{D,A}*K_{D,B}*K_{D,N}} \quad \text{(S18)}$$

The combination of equations S12-S18 with mass-balance equations S1-S3 results in the expression of $[A]_0$, $[B]_0$ and $[T]_0$ in the terms of [A], [B], [T] and dissociation constants.

$$[A]_0 = [A] + \frac{[A]*[B]}{K_{D,N}} + \frac{2[A]*[T]}{K_{D,A}} + \frac{[A]*[T]^2}{(K_{D,A})^2} + \frac{4[A]*[B]*[T]}{K_{D,A}*K_{D,B}} + \frac{4[A]*[B]*[T]*EM}{K_{D,A}*K_{D,B}*K_{D,N}} \quad \text{(S19)}$$

$$[B]_0 = [B] + \frac{[A]*[B]}{K_{D,N}} + \frac{2[B]*[T]}{K_{D,B}} + \frac{[B]*[T]^2}{(K_{D,B})^2} + \frac{4[A]*[B]*[T]}{K_{D,A}*K_{D,B}} + \frac{4[A]*[B]*[T]*EM}{K_{D,A}*K_{D,B}*K_{D,N}} \quad \text{(S20)}$$

$$[T]_0 = [T] + \frac{2[A]*[T]}{K_{D,A}} + \frac{2[B]*[T]}{K_{D,B}} + \frac{2[A]*[T]^2}{(K_{D,A})^2} + \frac{2[B]*[T]^2}{(K_{D,B})^2} + \frac{4[A]*[B]*[T]}{K_{D,A}*K_{D,B}} + \frac{4[A]*[B]*[T]*EM}{K_{D,A}*K_{D,B}*K_{D,N}} \quad \text{(S21)}$$

Equations S19-S21 were solved by using the Matlab (R2019a) function *fsolve*, yielding the free concentrations of A, B and T with the assigned values of $[A]_0$, $[B]_0$, $[T]_0$, $K_{D,A}$, $K_{D,B}$, $K_{D,N}$ and EM. Concentrations of the other complexes ([AB], [TA], [TB], $[T_2A]$, $[T_2B]$, [TABi] and [TABa]) were calculated using equations S12-S18. An example behavior of the system for a representative set of specific concentrations and values is shown in Supplementary Figure 2.

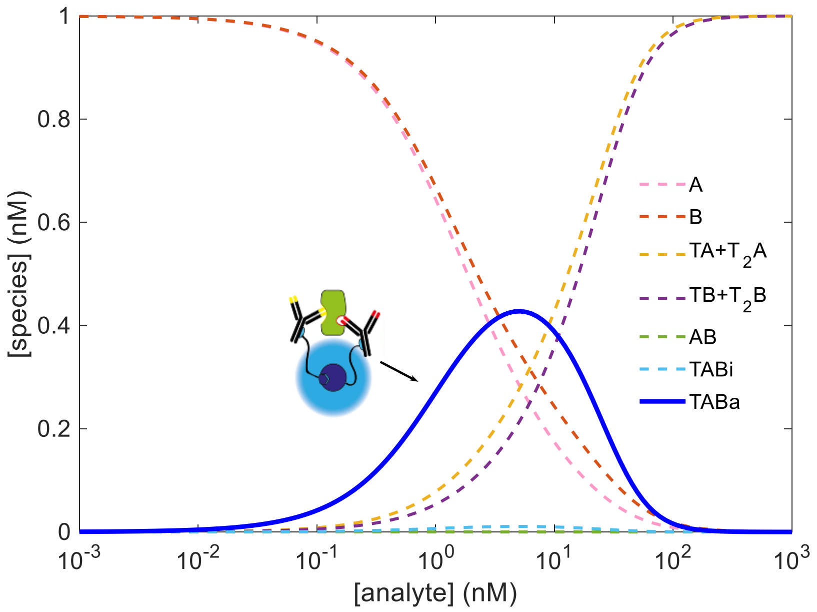

Supplementary Figure 2. Speciation plot illustrating the concentration of various species versus analyte concentration. Calculations were performed using $[A]_0 = [B]_0 = 1$ nM, $K_{D,A}$ =10 nM, $K_{D,B}$ = 15 nM, $K_{D,N}$ = 2.5 µM, EM = 100 µM.

To illustrate the dependence of the signal response on different variables, we performed simulations that varied the concentrations of antibody-conjugates, binding affinities and the effective molarity (EM) (Supplementary Figure 3). As shown in Supplementary Figure 3A, the response can be tuned by varying the concentration of the sensor components. The use of high concentrations of antibody conjugates shifts the “hook” of the response curve to the higher analyte concentrations. The sensor response is also affected by the interaction between the split NanoLuc fragments LB and SB (Supplementary Figure 3B). While a weaker interaction shows a strong attenuation of the luminescent ternary complex, a stronger interaction also results in a higher background signal in the absence of analyte. The sensitivity and detection range of the system can also be tuned by using antibodies with different affinity to their target (Supplementary Figure 3C). The use of antibodies with higher affinity leads to more sensitive sensors exhibiting a more intensive signal at low analyte concentrations. The luminescent signal is also highly affected by the EM which depends on the length and flexibility of the linkers and the distance that the linkers need to bridge in the ternary sandwich complex[1,4,5] (Supplementary Figure 3D). With a linker of 75 amino acids and a distance of 10 nm, we estimate an EM value between 1~10 µM[4]. Notably, the hook of the bell-shaped response curve only shows dependency on antibody affinities and sensor concentrations when they aresimilar to the dissociation constants $K_{D,A}$ and $K_{D,B}$. When the sensor concentrations $[A]_0$ and $[B]_0$ are substantially smaller than $K_{D,A}$ and $K_{D,B}$, the hook position is only affected by the antibody affinity to the target.

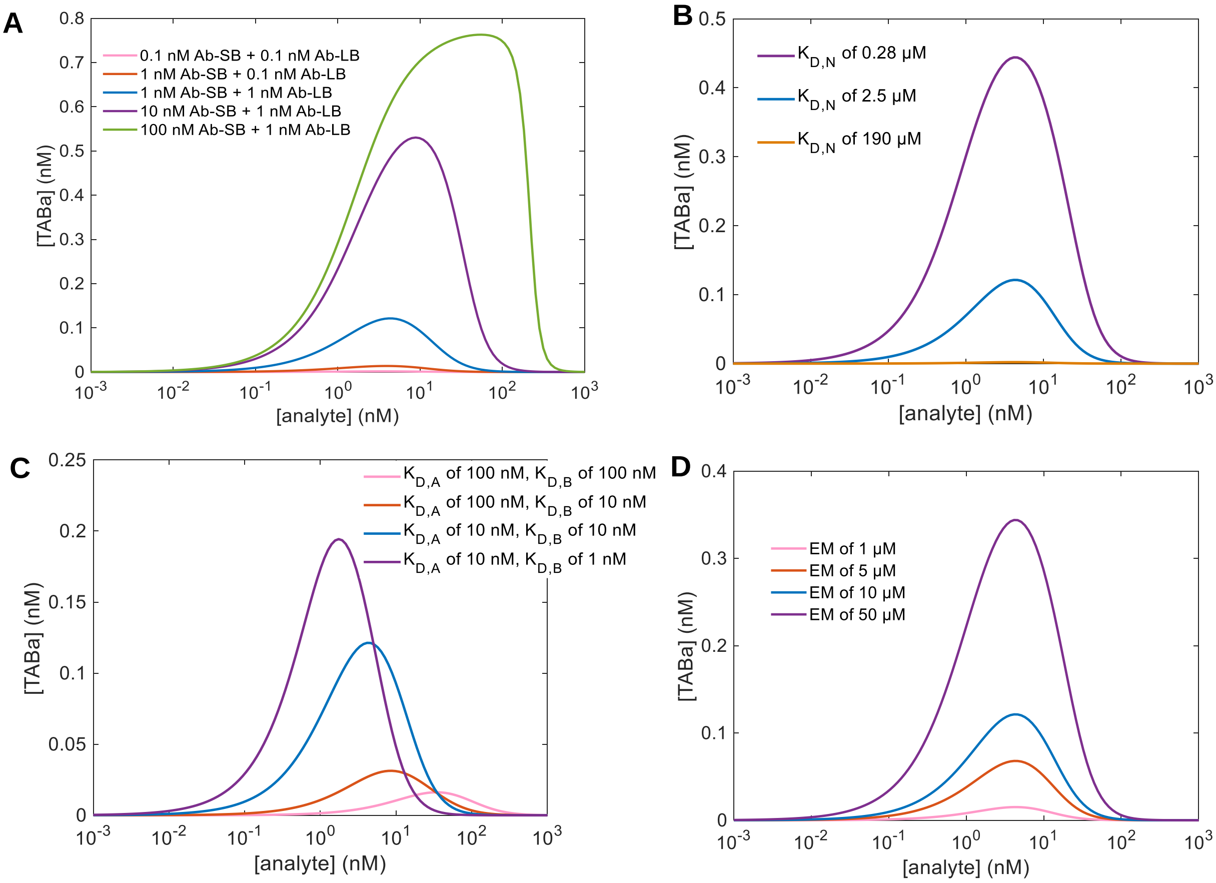

Supplementary Figure 3. Simulation of response curve for different parameters. (**A**) The response curves for different sensor concentrations using $K_{D,A}$ = $K_{D,B}$ = 10 nM, EM = 10 µM, $K_{D,N}$ = 2.5 µM. (**B**)The response curves for different split NanoLuc interaction affinities using $[A]_0$ = $[B]_0$ = 1 nM, $K_{D,A}$ = $K_{D,B}$ = 10 nM, EM = 10 µM. (**C**) The response curves for different antibody affinities using $[A]_0$ = $[B]_0$ = 1 nM, EM = 10 µM, $K_{D,N}$ = 2.5 µM. (**D**) The response curves with different EM values using $[A]_0$ = $[B]_0$ = 1 nM, $K_{D,N}$ = 2.5 µM, $K_{D,A}$ = $K_{D,B}$ = 10 nM.

We further fitted the experimental data to the model using the Matlab function *fitnlm* under the assumption that the sensor signal is proportional to the concentration of the active ternary complex (TABa), resulting in an estimation of the unknown parameters (i.e. $K_{D,A}$,$K_{D,B}$ and a scaling factor). After

subtraction of the background luminescence signal, the data of intensiometric cTnI assays was fitted to the model, yielding values of $K_{D,A}$ and $K_{D,B}$ of 533 and 15 nM, respectively when the EM is fixed at 10 µM (Supplementary Figure 4).

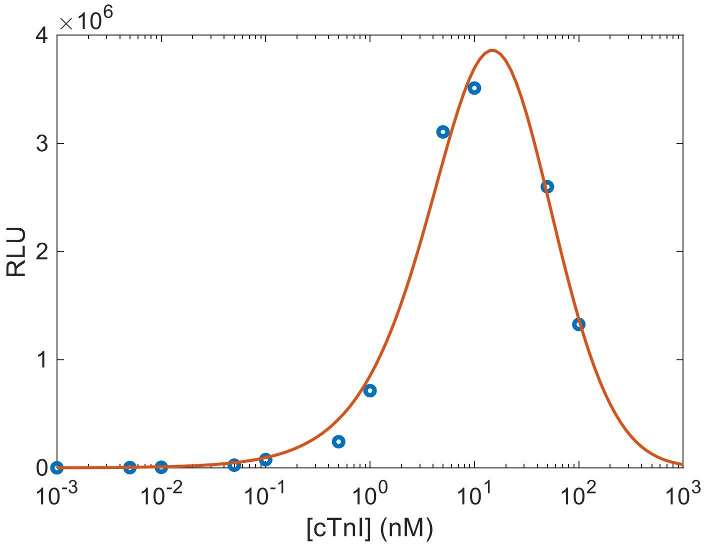

Estimated Coefficients:

	**Estimate**	**SE**
**$K_{D,A}$ (nM)**	533.34	15.658
**$K_{D,B}$ (nM)**	15.314	0.93664
**Scaling factor**	5.5315e+08	1.6853e-05

Number of observations: 11, Error degrees of freedom: 9

Root Mean Squared Error: 1.42e+05

R-Squared: 0.99, Adjusted R-Squared 0.989

F-statistic vs. zero model: 771, p-value = 8.64e-11

Supplementary Figure 4. Experimental data fitting to the model. The experimental data (blue circles) of the intensiometric assays of cTnI was fitted to the model using the Matlab function *fitnlm*, with the resultant non-linear fitting statistics provided below the figure. The experimental data (shown in Figure 2B) was subtracted by the background signal in the absence of cTnI. Nonlinear regression was performed using $[A]_0$ = 1 nM, $[B]_0$ = 1 nM, $K_{D,N}$ = 2.5 µM, EM = 10 µM, the initial $K_{D,A}$ = $K_{D,B}$ = 10 nM, scaling factor = 1×10^8. The fitted curve is plotted as a red solid line. An F-test was performed to test if the model significantly differs from a naïve model with only constant terms. The p-value was calculated from the upper tail of the underlying F-distribution.

Supplementary figures

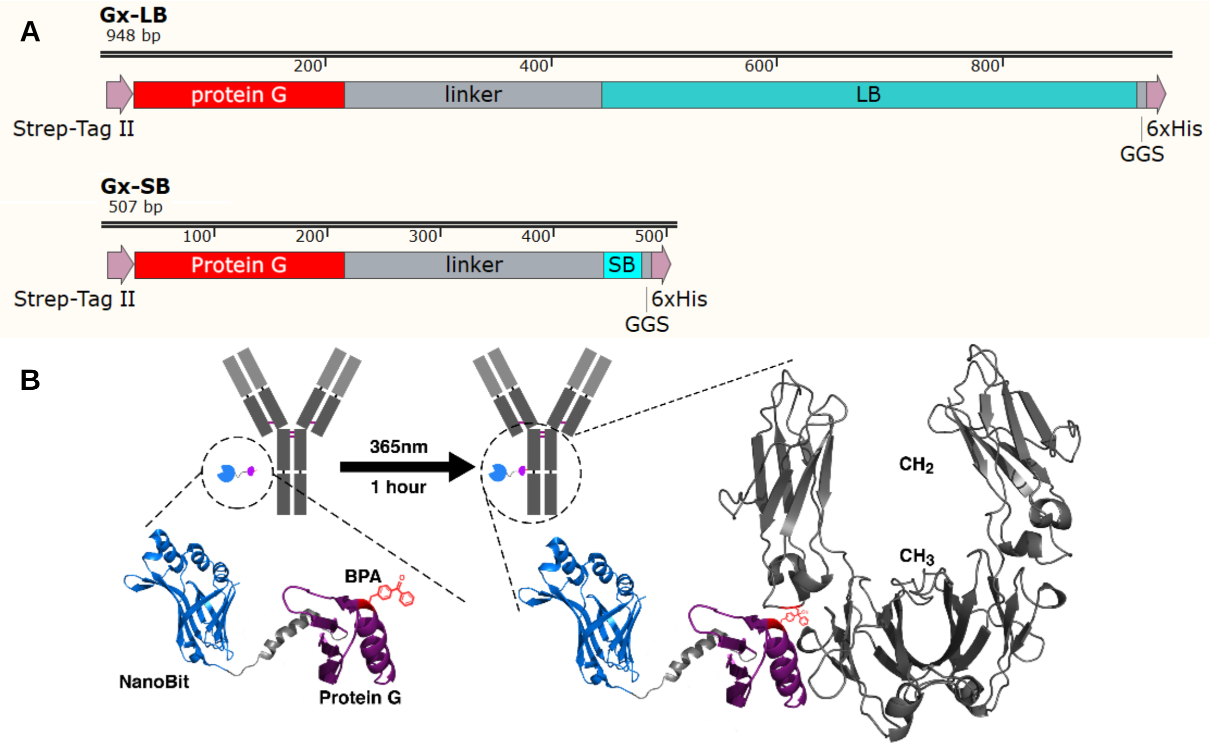

Supplementary Figure 5. Schematic representation of protein G-mediated photoconjugation. (**A**) Schematic representation of the constructs of Gx-LB and Gx-SB fusion proteins. A strep-tag and a His-tag are included at the N- and C-terminus, respectively, to facilitate the purification of the proteins. (**B**) Antibody conjugation with Gx-LB/SB by UV illumination. The protein G domain contains the unnatural amino acid BPA at position 24 (in red) and is fused to split NanoLuc (LB or SB) via a semiflexible linker consisting of two $(GGS)_6$ repeats and an $(EAAAK)_6$ α-helical block. The DNA and amino acid sequence of Gx-LB and Gx-SB are shown in Supplementary Figure 33 and 34.

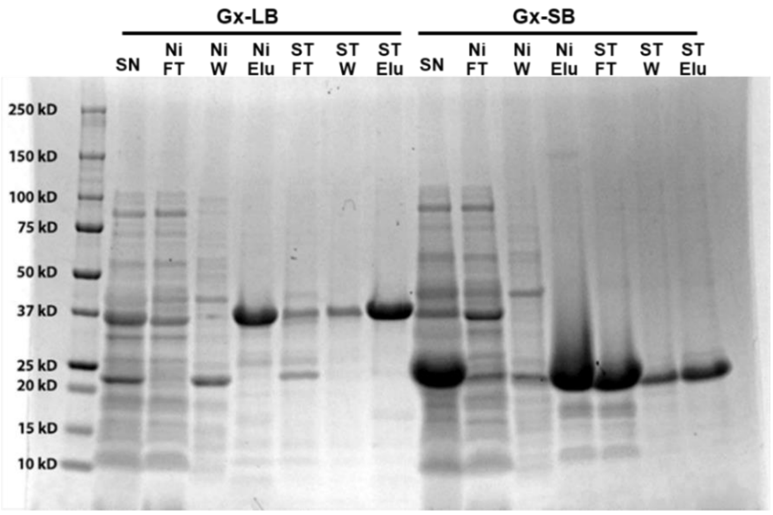

Supplementary Figure 6. SDS-PAGE analysis of Gx-LB and Gx-SB purification. The *E. coli*-expressed Gx-LB and Gx-SB fusion proteins were purified by using Ni^{2+} affinity chromatography and Strep-Tactin chromatography. SN: the supernatant of cell lysate; Ni FT/W/Elu: Ni^{2+} affinity chromatography flow through, wash and elution samples; ST FT/W/Elu: Strep-Tactin chromatography flow through, wash, and elution samples. Gel image depicts a representative image from n = 2 independent experiments, with similar results.

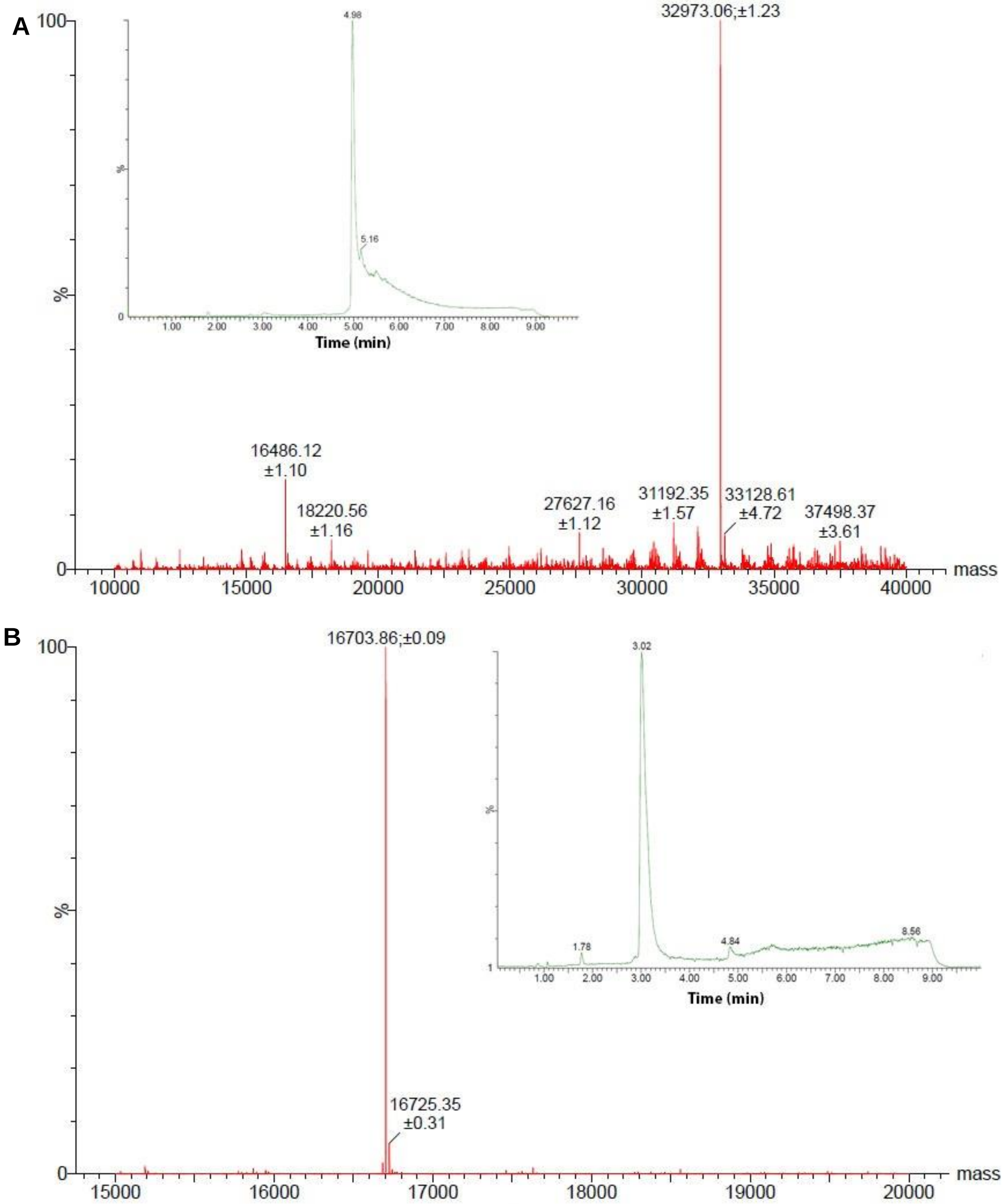

Supplementary Figure 7. ESI-QTOF mass spectra of Gx-LB and Gx-SB proteins. (**A**) Gx-LB (calculated mass lacking the N-terminal methionine: 32973Da). (**B**) Gx-SB (calculated mass lacking the N-terminal methionine: 16703Da). Inset shows the LC-MS chromatogram.

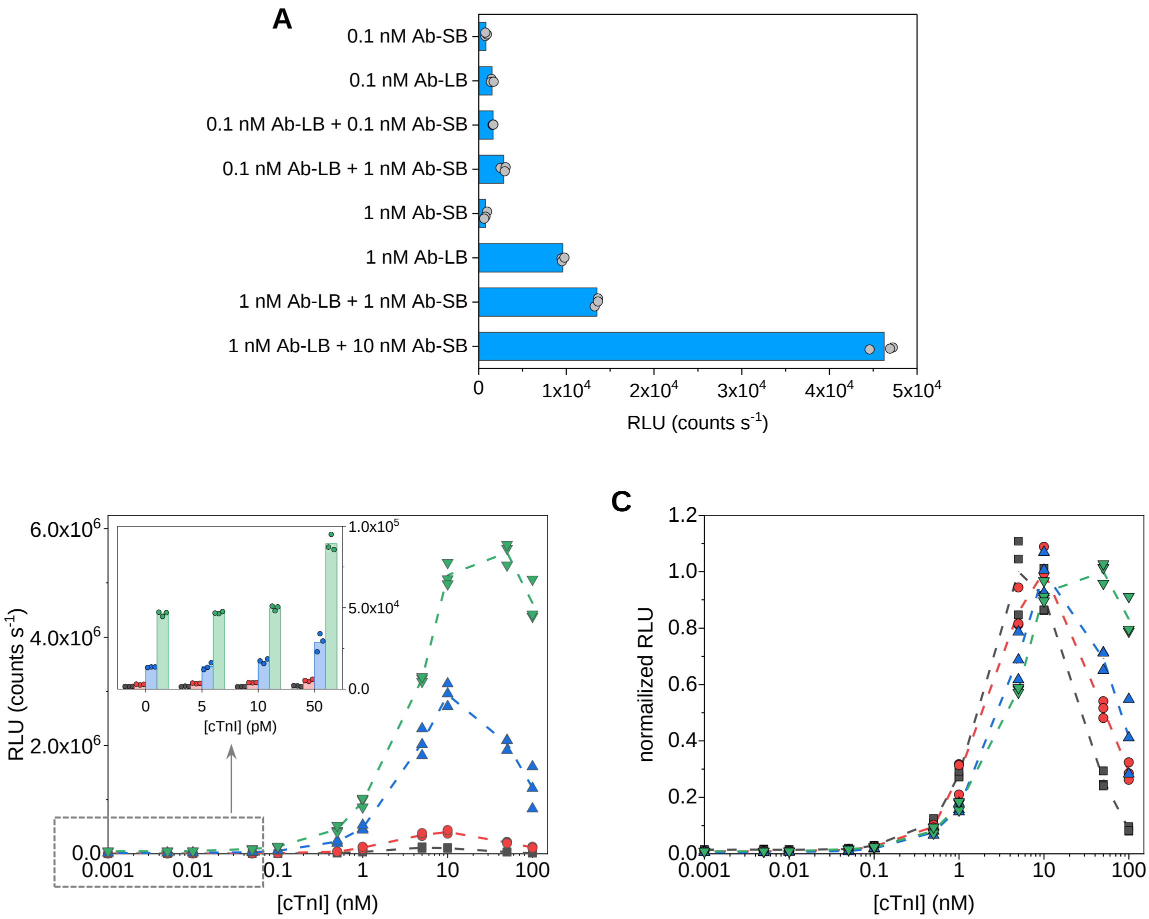

Supplementary Figure 8. CTnI dose-dependency using different sensor concentrations. (**A**) Background luminescence in the absence of analyte. While negligible luminescence was generated by Ab-SB, Ab-LB exhibited a larger background signal due to residual enzyme activity. When similar concentration of Ab-SB and Ab-LB were used in the assays, the background signal in the absence of analyte was primarily generated by Ab-LB. The use of higher absolute concentrations of Ab-SB resulted in an increased contribution of analyte-unbound binary complex to the background signal. (**B**) Intensiometric sensor response of cTnI in PBS buffer (pH 7.4, 0.1% (w/v) BSA) using (■) 0.1 nM 19C7-LB and 0.1 nM 4C2-SB; (●) 0.1 nM 19C7-LB and 1 nM 4C2-SB; (▲)1 nM 19C7-LB and 1 nM 4C2-SB; (▼) 1 nM 19C7-LB and 10 nM 4C2-SB. Sensor proteins were incubated with analyte for 30 minutes, followed by addition of NanoGlo substrate at 400-fold final dilution. Inset displays sensor behavior in the low-pM concentration regime as indicated by the dashed rectangle. LOD was calculated to be: 121 pM using 0.1 nM 19C7-LB and 0.1 nM 4C2-SB; 17 pM using 0.1 nM 19C7-LB and 1 nM 4C2-SB; 18 pM using 1 nM 19C7-LB and 1 nM 4C2-SB; 19 pM using 1 nM 19C7-LB and 10 nM 4C2-SB. (C) Normalized intensiometric sensor response of cTnI. The absolute signal intensity shown in Figure B was normalized to the highest intensities obtained respectively for the different sensor concentrations. Individual data points are represented as symbols, bars represent mean values, and dashed lines connect mean values (technical replicates, with n=3 independent preparations of the analyte). Source data are provided as a Source Data file.

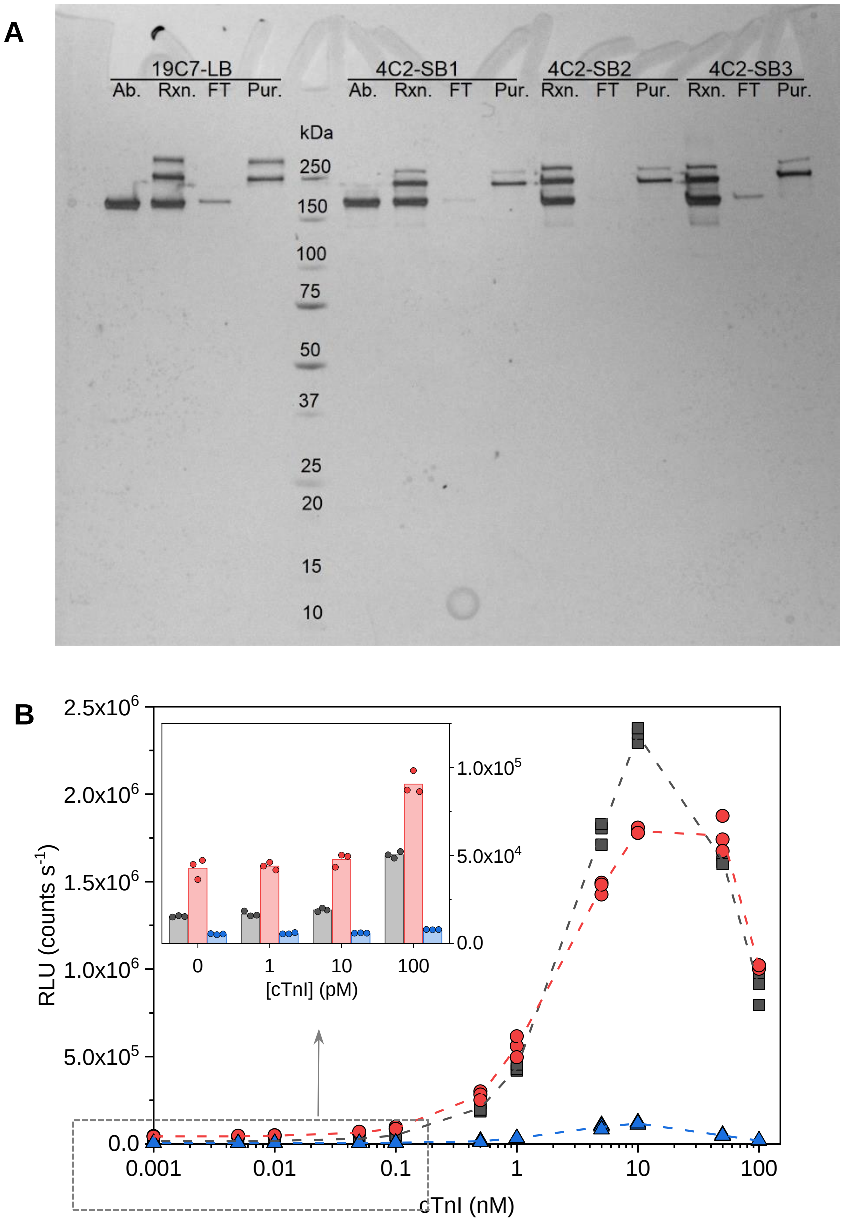

Supplementary Figure 9. CTnI dose-dependency using Gx-SB variants. (**A**) Non-reducing SDS-PAGE analysis (silver-stained) of anti-cTnI 4C2 antibody (Ab.) photoconjugated with different Gx-SB variants. Photoconjugation was performed using 1:2 molar ratio of anti-cTnI 4C2 to Gx-SB in PBS buffer (pH 7.4) for 1 hour. The photoconjugated products (Rxn.) were purified by Ni^{2+} affinity spin columns to remove the unconjugated antibodies (FT, flow through) resulting in purified antibody conjugates (Pur.). Gel image depicts a representative image from n = 3 independent experiments, with similar results. (**B**) Intensiometric assay of cTnI using 1 nM 19C7-LB and 1 nM 4C2-SB variants in PBS buffer (pH 7.4, 0.1% (w/v) BSA). (■) 4C2-SB with K_D of 2.5 μM; (●) 4CS-SB2 with K_D of 0.28 μM; (▲) 4C2-SB3 with K_D of 190 μM. Sensor proteins were incubated with analyte for 30 minutes, followed by addition of NanoGlo substrate at 400-fold final dilution. Inset displays sensor behavior in the low-pM concentration regime as indicated by the dashed rectangle. Error bars represent mean ± SD (n = 3). The slightly lower maximal signal observed using 4C2-SB2 (K_D of 0.28 μM) compared to that using 4C2-SB (K_D of 2.5 μM) was not expected based on the model simulation which predicted a higher signal intensity (Supplementary Figure 3B). We assume that the specific activity of the fully complemented NanoLuc might be different for the different SB variants and thus the observed luminescence is not directly proportional to the concentration of ternary luminescent complex TABa. Individual data points are represented as symbols, and dashed lines connect mean values (technical replicates, with n=3 independent preparations of the analyte). Source data are provided as a Source Data file.

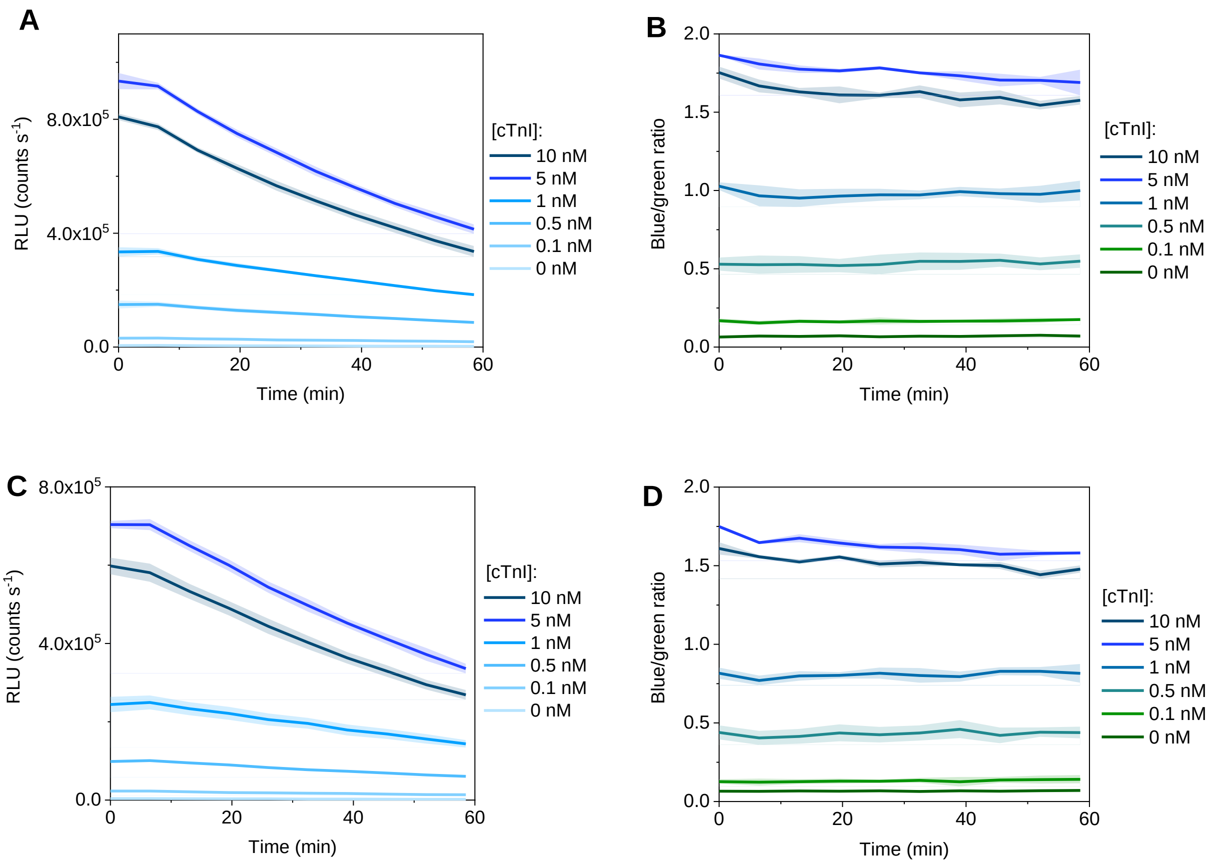

Supplementary Figure 10. Time dependence of intensiometric and ratiometric assays for cTnI. (**A, C**) Intensiometric assays using 1 nM 19C7-LB and 1 nM 4C2-SB in PBS buffer (pH 7.4, 0.1% (w/v) BSA). (**B, D**) Ratiometric assays with addition of 2 pM calibrator luciferase. Sensor proteins were incubated with different concentrations of cTnI for 30 minutes, followed by addition of NanoGlo substrate at 1000-fold (**A, B**) and 2000-fold (**C, D**) final dilution. Data are represented as mean ± SD (technical replicates, with n=3 independent preparations of the analyte). Source data are provided as a Source Data file.

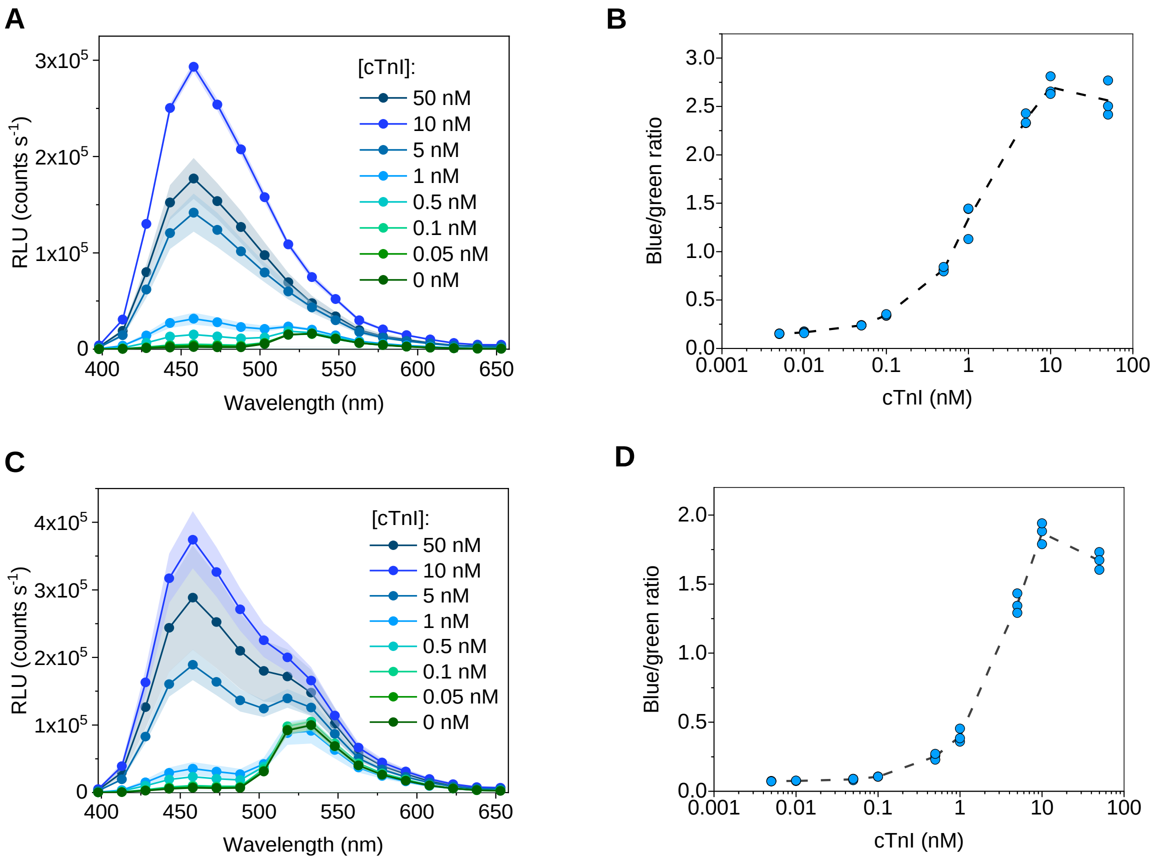

Supplementary Figure 11. Ratiometic assays of cTnI using different concentrations of calibrator luciferase. (**A**) Luminescence spectra at various concentrations of cTnI using 1 nM 19C7-LB, 1 nM 4C2-SB and 1 pM calibrator luciferase. (**B**) Sensor response curve for cTnI using 1 nM 19C7-LB, 1 nM 4C2-SB and 1 pM calibrator luciferase. (**C**) Luminescence spectra at various concentrations of cTnI using 1 nM 19C7-LB, 1 nM 4C2-SB and 5 pM calibrator luciferase. (**D**) Sensor response curve for cTnI using 1 nM 19C7-LB, 1 nM 4C2-SB and 5 pM calibrator luciferase. Assays were performed in PBS buffer (pH7.4, 0.1% (w/v) BSA). Sensor proteins were incubated with cTnI for 30 minutes, followed by addition of NanoGlo substrate at 400-fold final dilution. Data in A, C are represented as mean ± SD (technical replicates, with n=3 independent preparations of the analyte). In B, D individual data points are represented as circles, and dashed lines connect mean values (technical replicates, with n=3 independent preparations of the analyte). Source data are provided as a Source Data file.

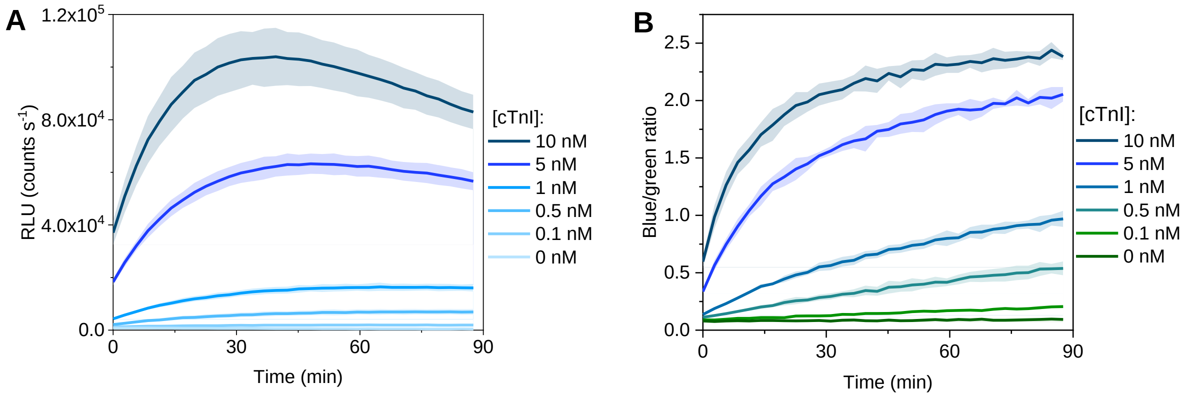

Supplementary Figure 12. Kinetics of intensiometric and ratiometric detection in a one-step assay for cTnI. (**A**) Intensiometric assays using 1 nM 19C7-LB and 1 nM 4C2-SB in PBS buffer (pH7.4, 0.1% (w/v) BSA) with NanoGlo substrate at 400-fold final dilution. (**B**) Ratiometric assays with addition of 2 pM calibrator luciferase. All components (sensor, calibrator, analyte and NanoGlo substrate) were added simultaneously at *t*=0, i.e. no pre-incubation of sensor proteins with analyte was involved. Data are represented as mean ± SD (technical replicates, with n=3 independent preparations of the analyte). Source data are provided as a Source Data file.

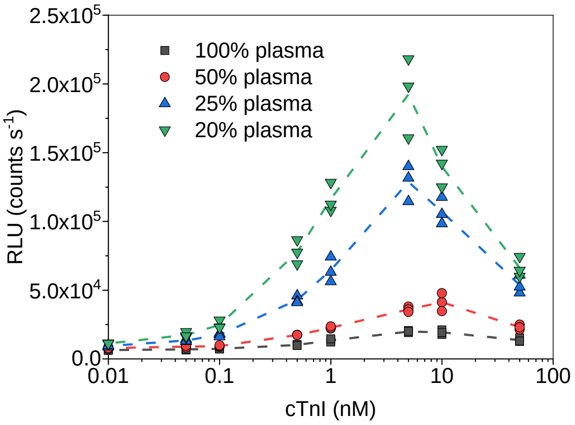

Supplementary Figure 13. Intensiometric assays of cTnI in human blood plasma. Assays were performed using 1 nM 19C7-LB and 1 nM 4C2-SB in 100%, 50%, 25% and 20% spiked human blood plasma (diluted in PBS buffer containing 0.1% (w/v) BSA). The plasma represents the final amount in assay mixtures. Sensor proteins were incubated with cTnI for 30 minutes, followed by addition of NanoGlo substrate at 400-fold final dilution. Individual data points are represented as symbols, and dashed lines connect mean values (technical replicates, with n=3 independent preparations of the analyte). Source data are provided as a Source Data file.

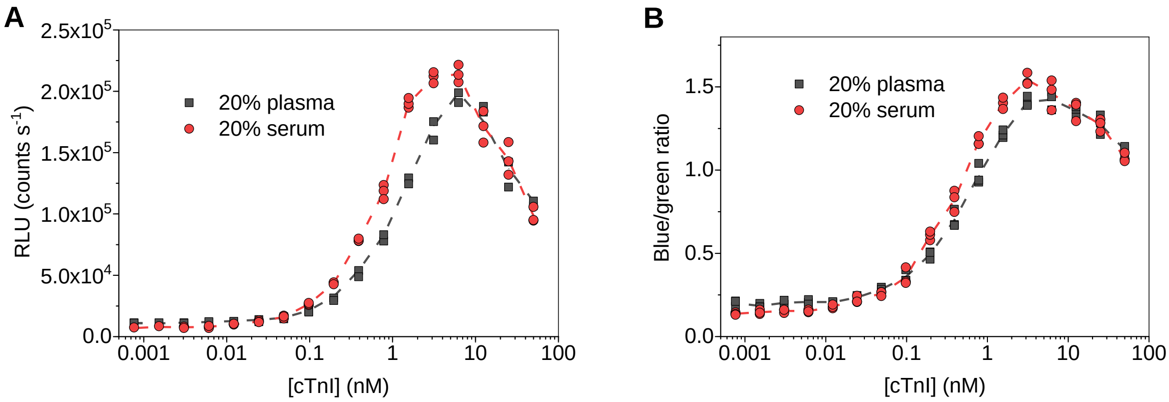

Supplementary Figure 14. Comparison of cTnI assays in human blood plasma and serum. (**A**) Intensiometric assays using 1 nM 19C7-LB and 1 nM 4C2-SB in 20% human blood plasma or 20% human blood serum (diluted in PBS buffer containing 0.1% (w/v) BSA). (**B**) Ratiometric assays with addition of 2 pM calibrator luciferase. The plasma and serum content represents the final amount in assay mixtures. Sensor proteins were incubated with cTnI for 30 minutes, followed by addition of NanoGlo substrate at 400-fold final dilution. Individual data points are represented as circles and squares, and dashed lines connect mean values (technical replicates, with n=3 independent preparations of the analyte). Source data are provided as a Source Data file.

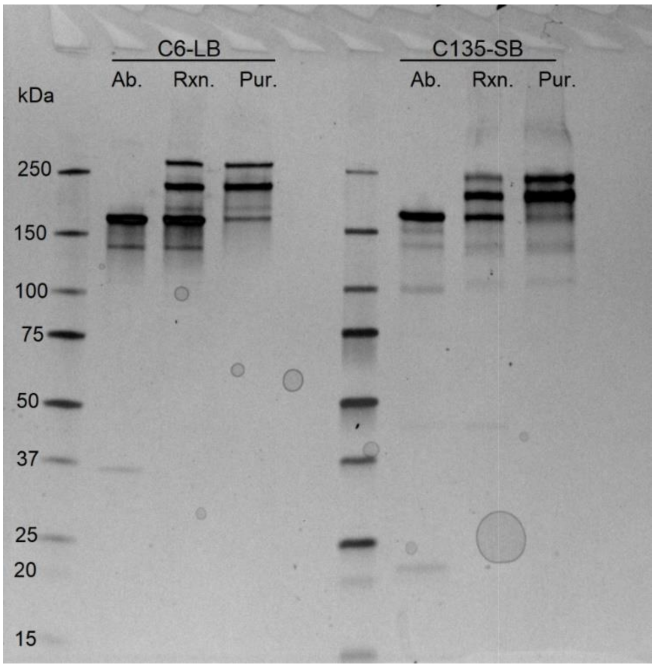

Supplementary Figure 15. Non-reducing SDS-PAGE analysis of photoconjugation of anti-CRP antibodies with Gx-LB and Gx-SB. Photoconjugation was performed using 1:1 molar ratio of anti-CRP antibodies (Ab.) C6 to Gx-LB, and 1:2 molar ratio of anti-CRP C135 to Gx-SB in PBS buffer (pH 7.4) for 2 hours. The photoconjugated products (Rxn.) were purified by Ni^{2+} affinity spin columns to remove non-conjugated antibodies resulting in purified antibody conjugates (Pur.). Gel image depicts a representative image from n = 2 independent experiments, with similar results.

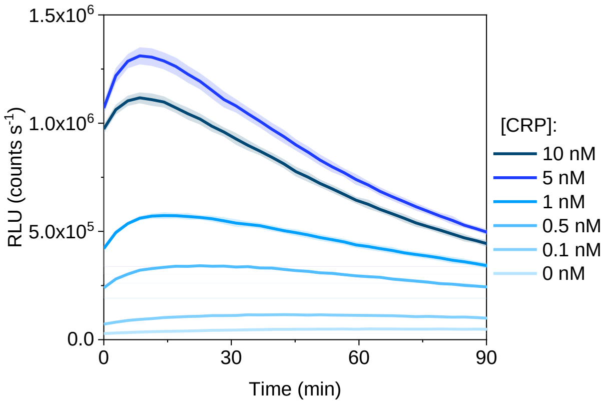

Supplementary Figure 16. Kinetics of intensiometric one-step assay for CRP. Assays were performed using 1 nM C6-LB and 10 nM C135-SB in PBS buffer (pH7.4, 0.1% (w/v) BSA) with NanoGlo substrate at 400-fold final dilution. All components (sensor, calibrator, analyte and NanoGlo substrate) were added simultaneously at *t*=0. Data are represented as mean ± SD (technical replicates, with n=3 independent preparations of the analyte). Source data are provided as a Source Data file.

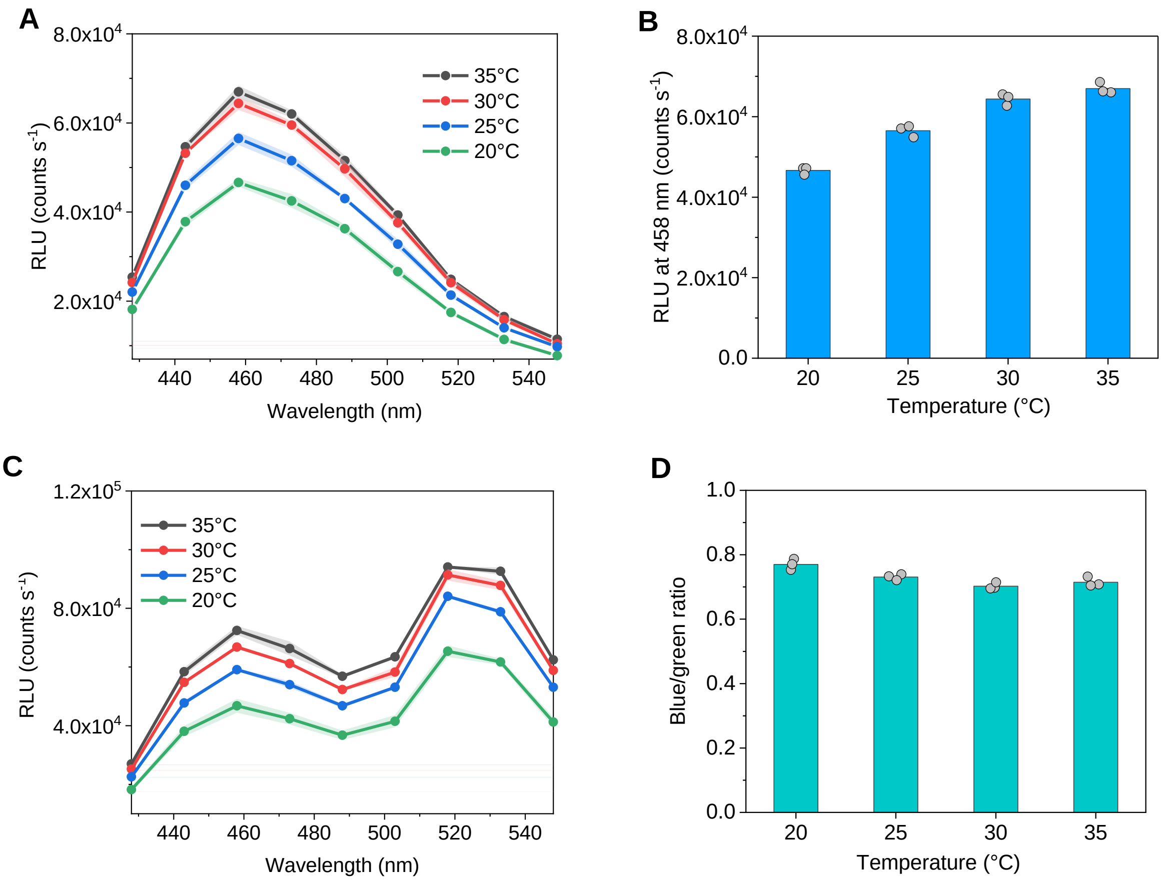

Supplementary Figure 17. Effect of temperature on NanoLuc and calibrator luciferase (mNG-NL) activity. (**A, B**) Luminescence spectra of 5 pM NanoLuc at different temperatures. (**C, D**) Luminescence spectra and emission ratio of a mixture of 5 pM NanoLuc and 5 pM calibrator luciferase at different temperatures. Assay mixtures in PBS buffer (pH 7.4, 0.1% (w/v) BSA) were incubated at indicated temperatures, followed by addition of NanoGlo substrate at 400-fold final dilution. Data in **A, C** are represented as mean ± SD (technical triplicates). In **B, D** individual data points are represented as circles, and bars represent mean values (technical replicates). Source data are provided as a Source Data file.

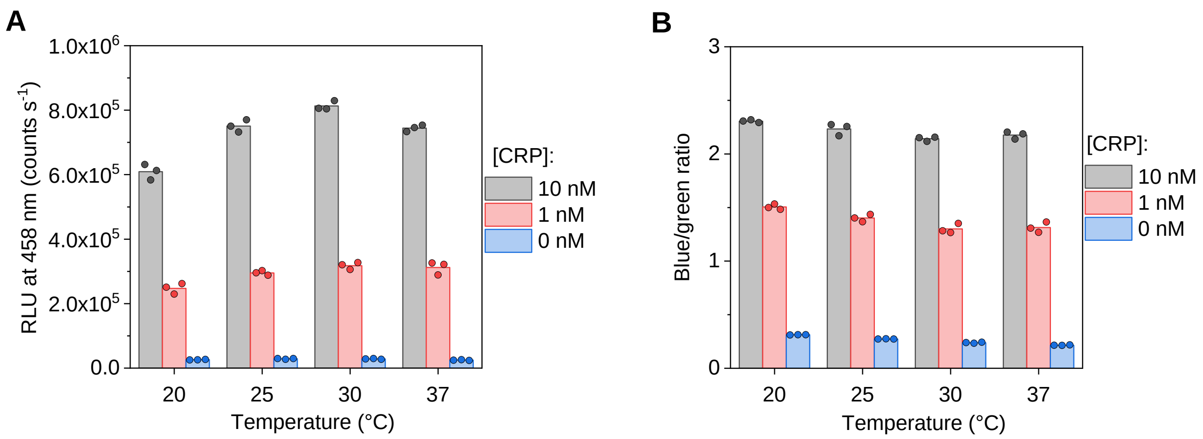

Supplementary Figure 18. Effect of temperature on intensiometric and ratiometric sensor output of the CRP assay. (**A**) Luminescence at 458 nm of intensiometric assays using 1 nM C6-LB and 10 nM C135-SB in PBS buffer (pH 7.4, 0.1% (w/v) BSA) at indicated temperatures. (**B**) Emission ratio of ratiometric assays with addition of 2 pM calibrator luciferase. Sensor proteins were incubated with different concentrations of CRP for 1 hour, followed by addition of NanoGlo substrate at 1000-fold final dilution. Individual data points are represented as circles, and bars represent mean values (technical replicates, with n=3 independent preparations of the analyte). Source data are provided as a Source Data file.

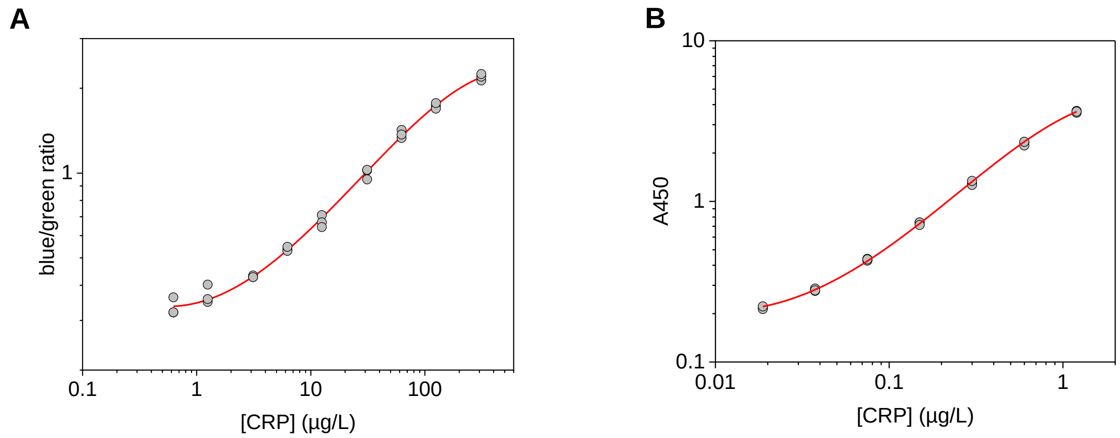

Supplementary Figure 19. Calibration of RAPPID and ELISA assays for CRP. (**A**) Calibration curve of the RAPPID assay for CRP. The CRP-standards were prepared by diluting the CRP (Hytest) in PBS buffer (pH7.4) containing 0.1% (w/v) BSA and measured three times using 1 nM C6-LB, 10 nM C135-SB and 2 pM calibrator luciferase. Sensor proteins were incubated with CRP for 30 minutes, followed by addition of NanoGlo substrate at 400-fold final dilution. The concentrations on the X-axis represent final CRP concentrations in the assay mixture. Individual data points are represented as circles (n = 3). A 4 parameter polynomial curve was fit through the data on a log blue-to-green ratio/log CRP concentration plot. The test samples were prepared by spiking different concentrations of CRP in pooled human blood plasma and subsequently diluting 1:50 in PBS buffer (pH7.4) containing 0.1% (w/v) BSA. Each pre-diluted sample was measured four times by using RAPPID. (**B**) Calibration curve of ELISA for CRP. A human C-reactive protein ELISA kit (KHA0031, ThermoFisher) was used according to the manufacturer's instructions. Standards were prepared by serially diluting the CRP (Hytest) in Standard Diluent Buffer and test samples were prepared in pooled human blood plasma and diluted 1:20000 with Standard Diluent Buffer. Standards and pre-diluted test samples were incubated 2 hours at 37°C in 96-well plates coated with capture antibody provided with the kit. Each standard was measured three times and each test sample was measured four times. After 4 washing cycles with wash buffer, the anti-human CRP antibody-Biotin conjugate was added and incubated for 1 hour at room temperature. After 4 additional wash steps, Streptavidin-HRP solution was added and incubated for 30 minutes at room temperature. Subsequent to the last 4 wash cycles, the Stabilized Chromogen was added and incubated for 30 minutes at room temperature protected from light. The reaction was stopped by adding Stop Solution, turning the solution from blue to yellow. Absorbance was read out on a Tecan Infinite F500 plate reader at 450 nm. Individual data points are represented as circles (technical replicates, with n=3 independent preparations of the analyte). According to the manufacturer's instruction, a 4 parameter curve was fit through the data on a log absorbance/log CRP concentration plot. Source data are provided as a Source Data file.

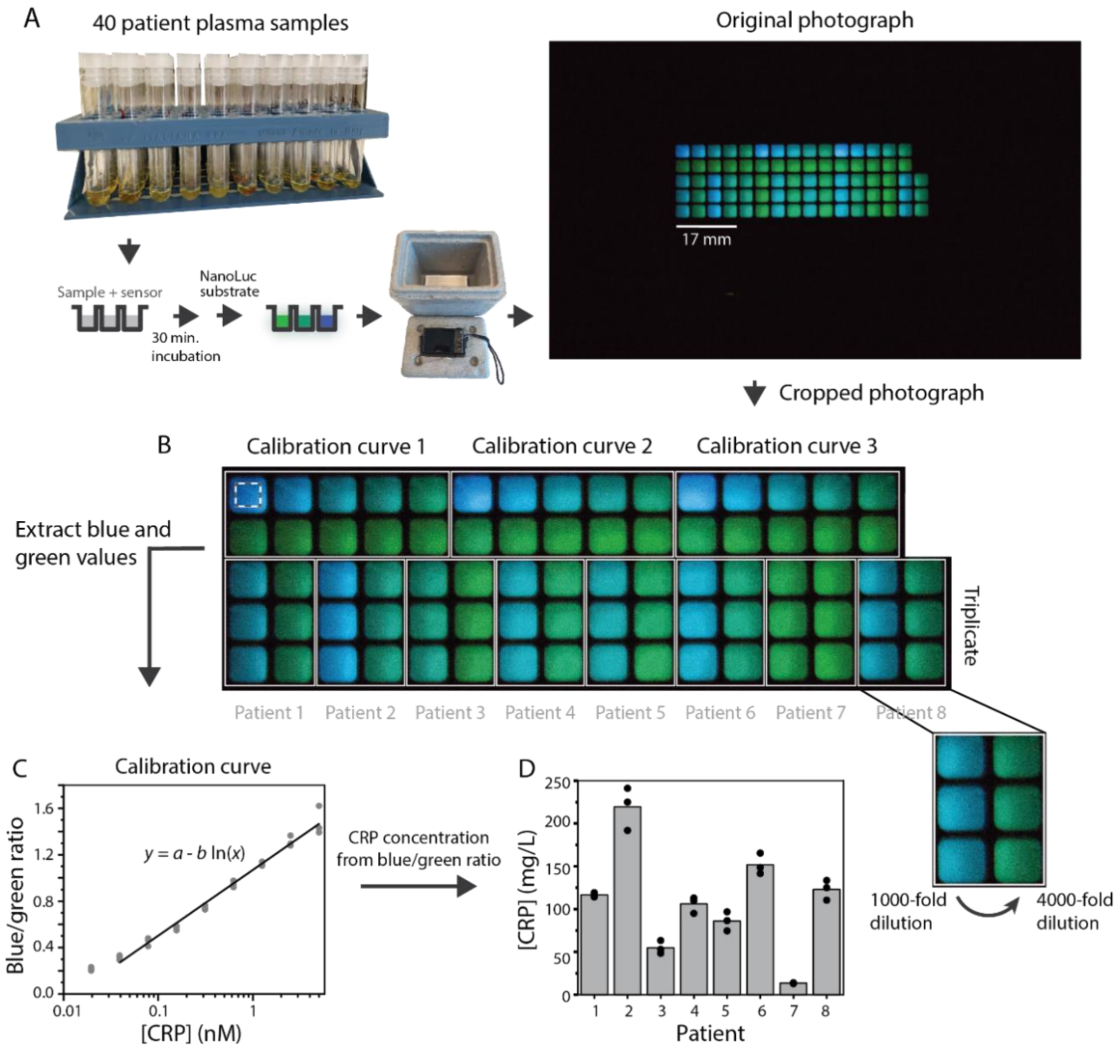

Supplementary Figure 20. Quantification of CRP in patient plasma samples. (A) Preparation of the 40 CRP patient plasma samples, measured in 5 individual runs with independent calibration curves, divided over 2 days. For each run, the plasma samples were diluted both 1,000-fold and 4,000-fold (in triplicate) in PBS buffer (pH 7.4, 0.1% (w/v) BSA) and mixed with 1 nM C6-LB, 10 nM anti-C135-SB and 14 pM calibrator luciferase. For calibration, the CRP-standards of 0 nM - 5 nM were prepared freshly for each run in triplicate in PBS buffer (pH 7.4, 0.1% (w/v) BSA) and mixed with sensor proteins at the same concentrations as those used for the patient samples. Both the patient samples and the calibration curves had a total volume of 20 µL and were incubated in a non-treated white Thermo Scientific™ 384 well plate (Cat. no 262360). After 30 minutes of incubation at room temperature, NanoGlo substrate (Promega, N1110) was added at a final dilution of 1000-fold. Directly after the addition of substrate, the plate was placed in a box and a photograph was taken with a digital camera (Sony DSC-RX100) with an exposure time of 10 seconds and an ISO value of 6400. **(B)** Cropped photograph of the calibration curves and the 1,000-fold and 4,000-fold diluted CRP patient samples. Photographs were analyzed in ImageJ 1.51j8 software[6] by retracting the intensity of the green- and blue light in a predefined square, in the middle of each well. **(C)** Calibration curve of the CRP-RAPPID assay. The known log CRP concentration in the calibration curves were plotted against the retracted blue to green ratios and a linear curve was fit through the linear part of the data. **(D)** Quantification of CRP levels in 8 patient samples within one run. The fit through the calibration curve in C was used to translate the blue/green ratios of the patient samples to CRP concentrations. The 1,000-fold dilution and 4,000-fold dilution of the patient samples was used for CRP concentrations below 60 mg/L and above 60 mg/L, respectively. In **C, D** individual data points are represented as circles, bars represent mean values (technical replicates, with n=3 independent preparations of the analyte). Source data of panels **C**, **D** and Fig. 3f are provided as a Source Data file.

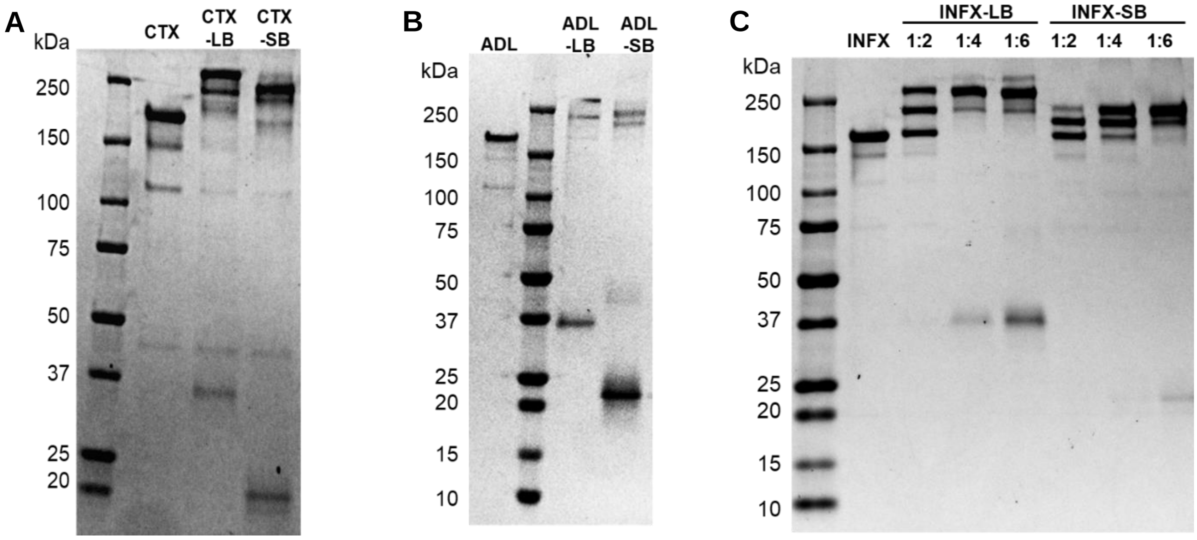

Supplementary Figure 21. Non-reducing SDS-PAGE analysis of therapeutic antibodies photoconjugated with Gx-LB and Gx-SB. (**A**) Cetuximab (CTX) photoconjugated with Gx-LB at 1:4 molar ratio, and with Gx-SB at 1:6 molar ratio. (**B**) Adalimumab (ADL) photoconjugated with Gx-LB at 1:4 molar ratio, and with Gx-SB at 1:6 molar ratio. (**C**) Infliximab (INFX) photoconjugated with Gx-LB at 1:2, 1:4, 1:6 molar ratio, and with Gx-SB at 1:2, 1:4, 1:6 molar ratio. Each photoconjugation reaction was performed in PBS buffer (pH 7.4) for 2 hours. The INFX-LB generated at 1:4 molar ratio and INFX-SB at 1:6 molar ratio were used in the RAPPID assay in Figure 4B. The generated antibody conjugates were directly used in the RAPPID assays without further purification. Gel image depicts an image from n = 1 independent experiments.

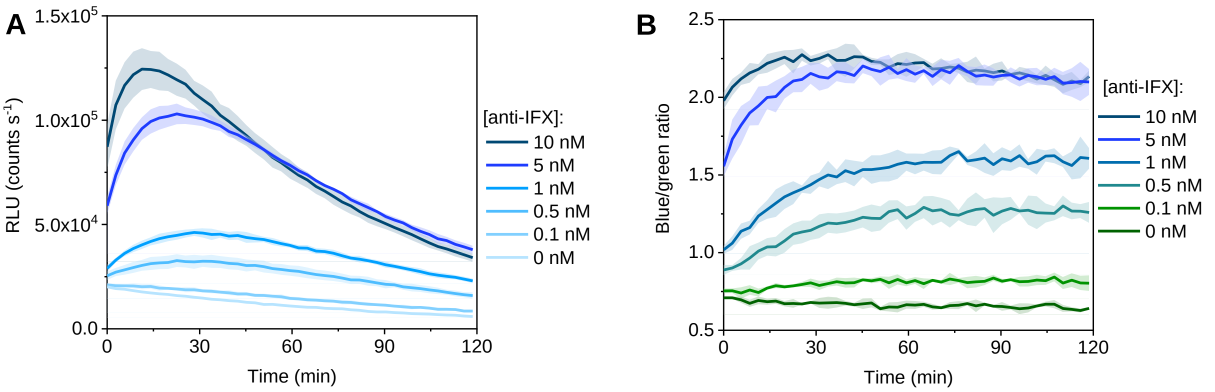

Supplementary Figure 22. Kinetics of intensiometric and ratiometric one-step assays for anti-infliximab. (**A**) Intensiometric assays using 1 nM INFX-LB and 1 nM INFX-SB in PBS buffer (pH 7.4, 0.1% (w/v) BSA) with NanoGlo substrate at 1000-fold final dilution. (**B**) Ratiometric assays with addition of 2 pM calibrator luciferase. All components (sensor, calibrator, analyte and NanoGlo substrate) were added simultaneously at *t*=0. Data are represented as mean ± SD (technical replicates, with n=3 independent preparations of the analyte). Source data are provided as a Source Data file.

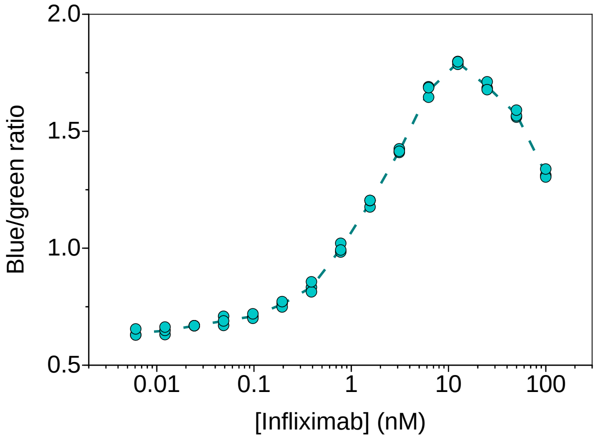

Supplementary Figure 23. RAPPID assays for infliximab using anti-infliximab conjugated sensor proteins. Assays were performed using 10 nM anti-INFX-LB and 10 nM anti-INFX-SB and 50 pM calibrator luciferase in 10% blood plasma. Sensor proteins were incubated with infliximab for 20 minutes, followed by addition of NanoGlo substrate at 500-fold final dilution. Individual data points are represented as circles, and dashed line connects mean values (technical replicates, with n=3 independent preparations of the analyte). Source data are provided as a Source Data file.

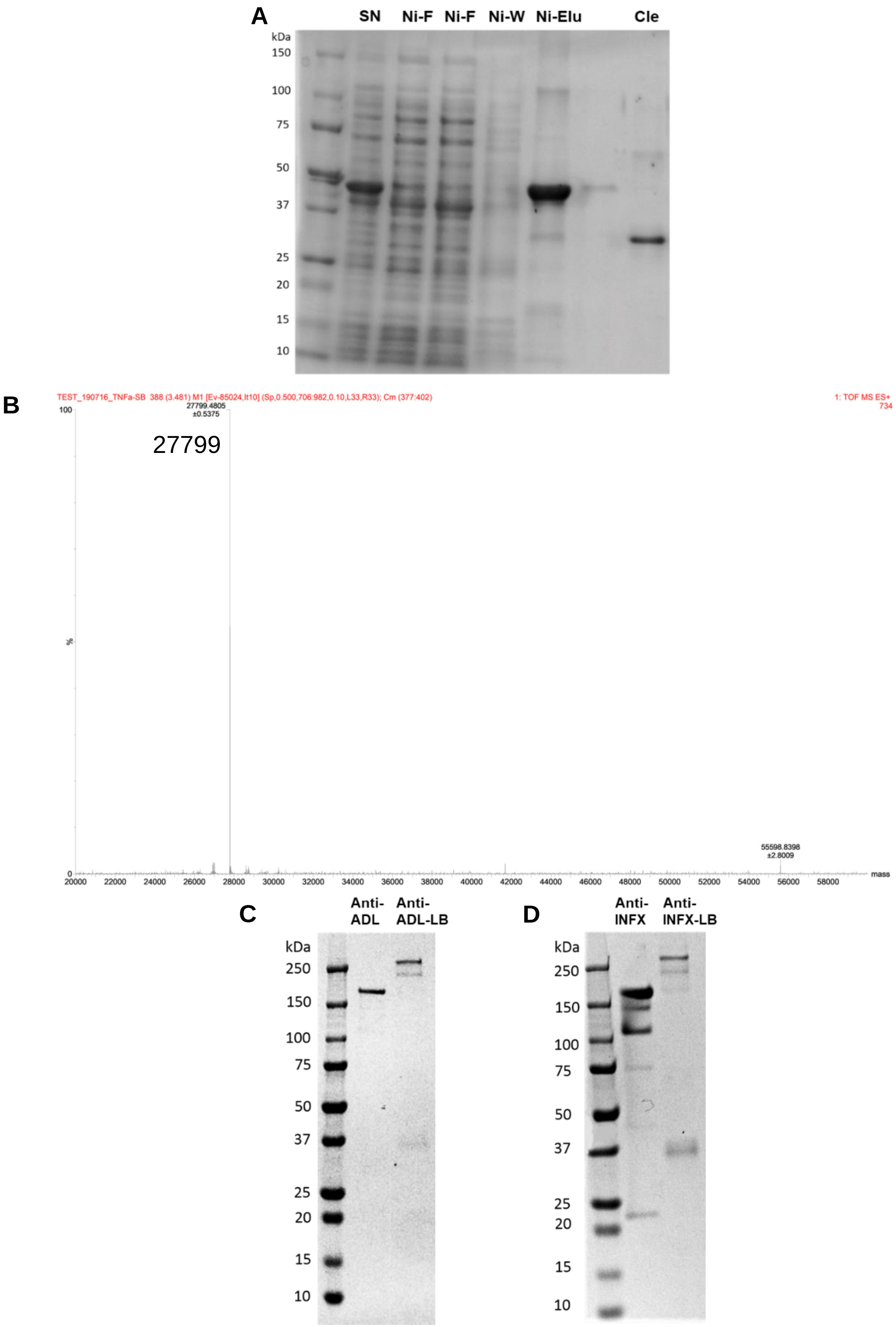

Supplementary Figure 24. SDS-PAGE and QTOF analysis of TNFα-SB and anti-antibodies photoconjugated with Gx-LB. (**A**) Purification of TNFα-SB. The *E. coli*-expressed TNFα-SB fusion proteins were purified by using Ni^{2+} affinity chromatography and the SUMO-tag was subsequently cleaved and removed by a second Ni^{2+} affinity chromatography. SN: the supernatant of cell lysate; Ni FT/W/Elu: Ni^{2+} affinity chromatography flow through, wash and elution samples; Cle: pure TNFα-SB after SUMO-tag cleavage. (**B**) ESI-QTOF mass spectrum of TNFα-SB (calculated mass with disulfide bond formed between two cysteine residues: 27799 Da). (**C**) Anti-adalimumab/TNFα monoclonal antibody (HCA207, BioRad) (anti-ADL) photoconjugated with Gx-LB at 1:4 molar ratio. (**D**) Anti-

infliximab (HCA213, BioRad) (anti-INFX) photoconjugated with Gx-LB at 1:4 molar ratio. Each photoconjugation was performed in PBS buffer (pH 7.4) for 2 hours. The generated anti-ADL-LB and anti-INFX-LB conjugates were directly used in the RAPPID assays without further purification. Gel image depicts an image from n = 1 independent experiments.

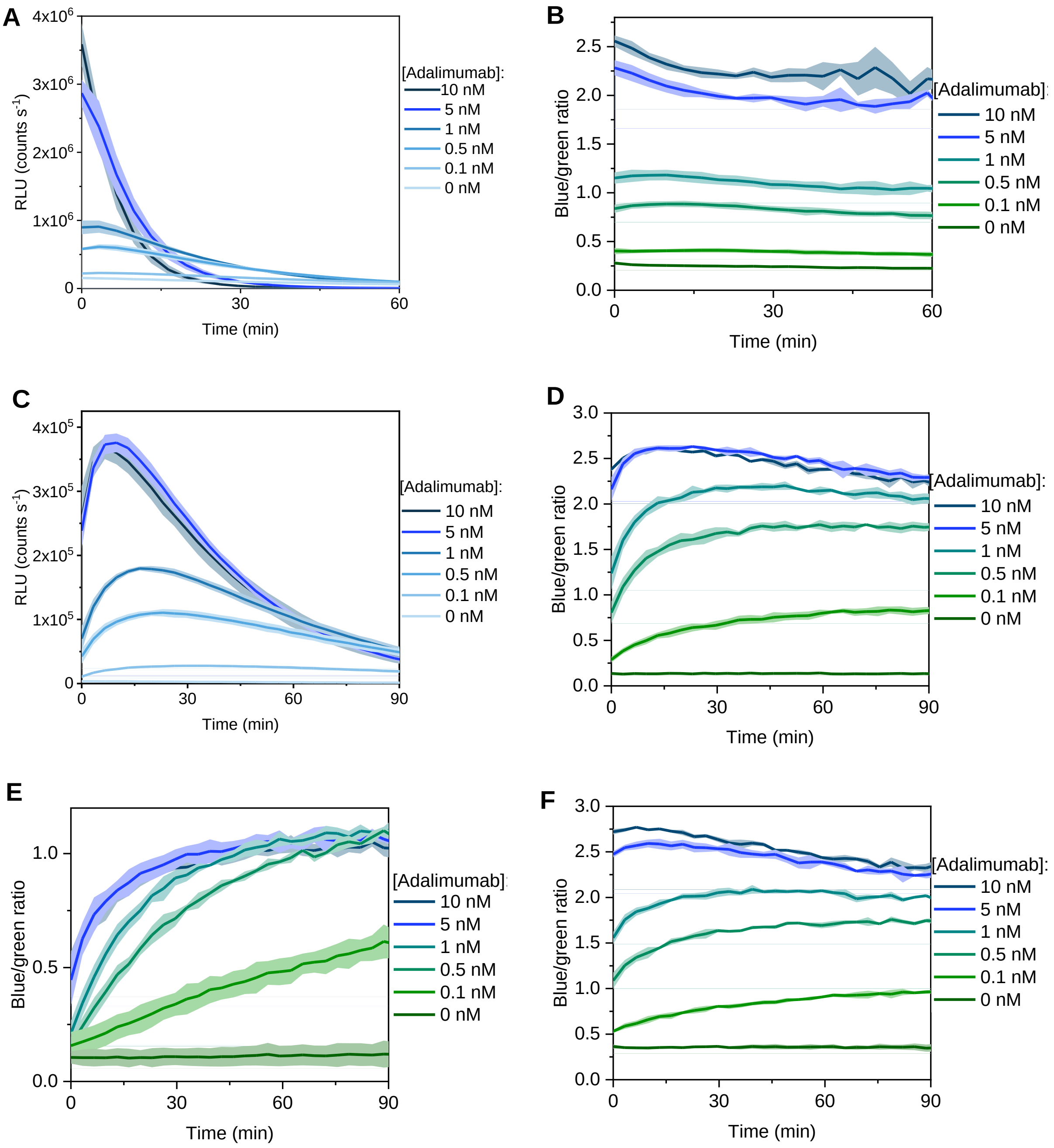

Supplementary Figure 25. Kinetics of the intensiometric and ratiometric one-step assays for Adalimumab. Assays were performed in PBS buffer (pH 7.4, 0.1% (w/v) BSA) containing different concentrations of sensor proteins: (**A, B**) 10 nM anti-ADL-LB and 100 nM TNFα-SB; (**C, D**) 1 nM anti-ADL-LB and 10 nM TNFα-SB; (**E**) 1 nM anti-ADL-LB and 1 nM TNFα-SB; (**F**) 1 nM anti-ADL-LB and 100 nM TNFα-SB. Calibrator mNG-NL of 50 pM was added in ratiometric assays (**B, D, E** and **F**). NanoGlo substrate was added at 1000-fold final dilution in all assays in this figure. All components (sensor, calibrator, analyte and NanoGlo substrate) were added simultaneously at *t*=0. Data are represented as mean ± SD (technical replicates, with n=3 independent preparations of the analyte). Source data are provided as a Source Data file.

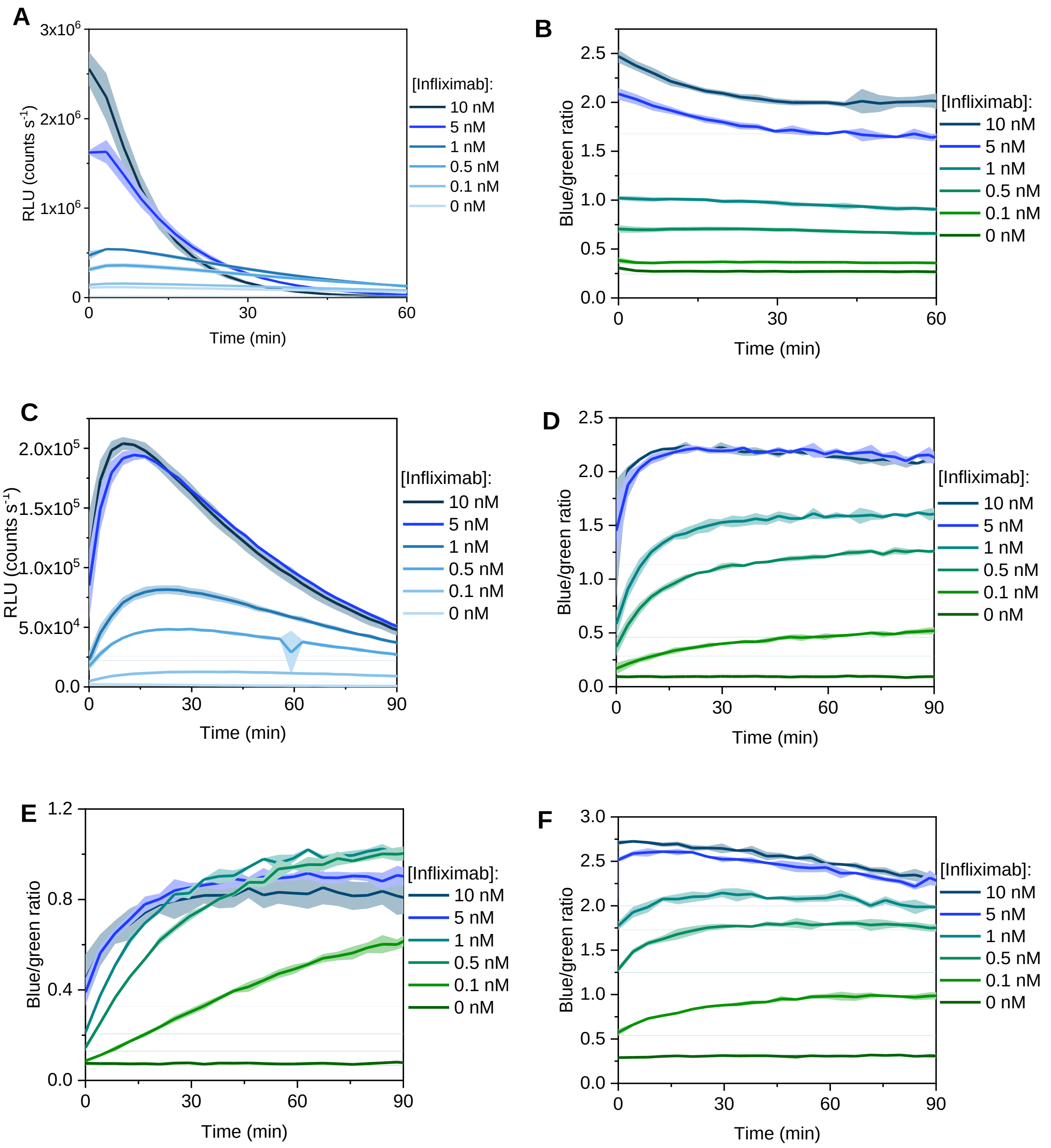

Supplementary Figure 26. Kinetics of the intensiometric and ratiometric one-step assays for Infliximab. Assays were performed in PBS buffer (pH 7.4, 0.1% (w/v) BSA) containing different concentrations of sensor proteins: (**A, B**) 10 nM anti-INFX-LB and 100 nM TNFα-SB; (**C, D**) 1 nM anti-INFX-LB and 10 nM TNFα-SB; (**E**) 1 nM anti-INFX-LB and 1 nM TNFα-SB; (**F**) 1 nM anti-INFX-LB and 100 nM TNFα-SB. Calibrator mNG-NL of 50 pM was added in ratiometric assays (**B, D, E** and **F**). NanoGlo substrate was added at 1000-fold final dilution in all assays in this figure. All components (sensor, calibrator, analyte and NanoGlo substrate) were added simultaneously at *t*=0. Data are represented as mean ± SD (technical replicates, with n=3 independent preparations of the analyte). Source data are provided as a Source Data file.

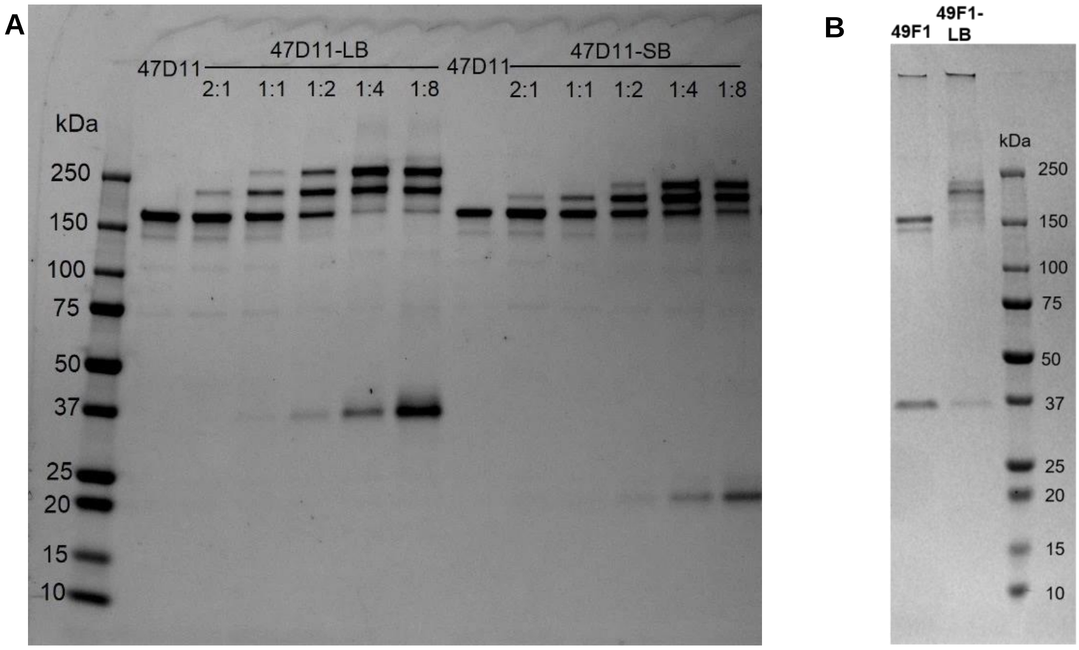

Supplementary Figure 27. Non-reducing SDS-PAGE analysis of anti-SARS-Cov-2 antibodies photoconjugated with Gx-LB and Gx-SB. (**A**) 47D11 photoconjugated to Gx-LB and Gx-SB using different molar ratio of antibody to Gx-LB/SB. (**B**) 49F1 photoconjugated to Gx-LB using 1:4 molar ratio of antibody to Gx-LB. Photoconjugation was performed in PBS buffer (pH7.4) for 2 hours. The generated antibody conjugates were directly used in the RAPPID assays without further purification. Gel image depicts an image from n = 1 independent experiments.

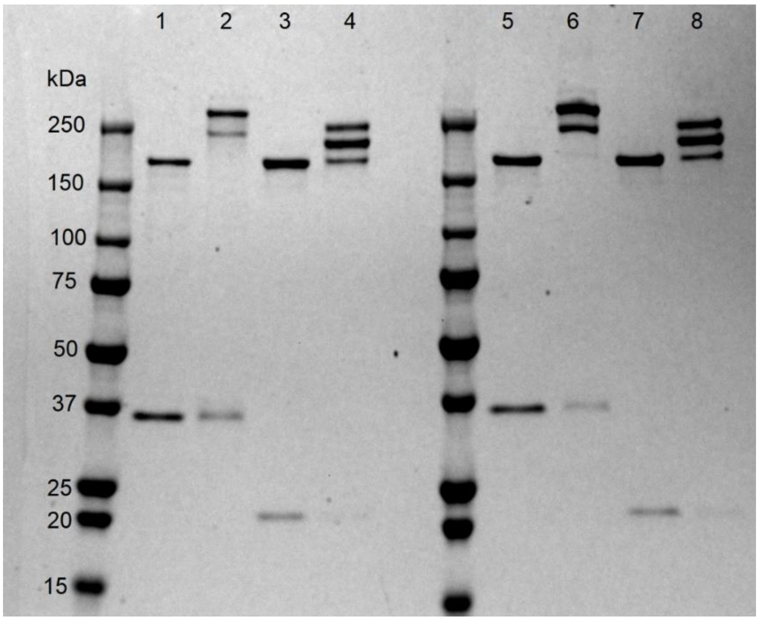

Supplementary Figure 28. Non-reducing SDS-PAGE analysis of commercial SARS-Cov-2 antibodies photoconjugated with Gx-LB and Gx-SB. Commercial SARS-Cov-2 antibodies D001, D002, D003 and D004 were ordered from Sino Biological (Beijing, China). Photoconjugation was performed using 1:4 molar ratio of antibody to Gx-LB/SB in PBS buffer (pH7.4) for 2 hours. Lane 1, mixture of D001 and Gx-LB; Lane 2, photoconjugated D001-LB; Lane 3, mixture of D003 and Gx-SB; Lane 4, photoconjugated D003-SB; Lane 5, mixture of D002 and Gx-LB; Lane 6, photoconjugated D002-LB; Lane 7, mixture of D004 and Gx-SB; Lane 8, photoconjugated D004-SB. The generated antibody conjugates were directly used in the RAPPID assays without further purification. Gel image depicts an image from n = 1 independent experiments.

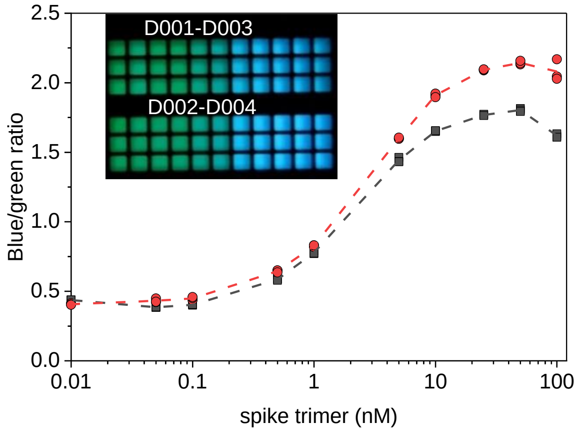

Supplementary Figure 29. Ratiometric assays of spike protein using commercial SARS-COV-2 antibodies conjugated with Gx-SB and Gx-LB. Assays were performed using 1 nM D001-LB with 10 nM D003-SB (■) or 1 nM D002-LB with 10 nM D004-SB (●) in PBS buffer (pH 7.4, 0.1% (w/v) BSA) spiked with 10 pM calibrator luciferase. The antibody conjugates (Ab-LB and Ab-SB) were respectively mixed with 200-fold excess of cetuximab (200 nM and 2 µM, respectively) to capture the non-conjugated Gx-LB and Gx-SB in the solution. The mixed sensor proteins were then incubated with spike protein for 1 hour, followed by addition of NanoGlo substrate at 400-fold final dilution. Individual data points are represented as circles and squares, and dashed lines connect mean values (technical replicates, with n=3 independent preparations of the analyte). Insert: photographs taken by a digital camera. Source data are provided as a Source Data file.

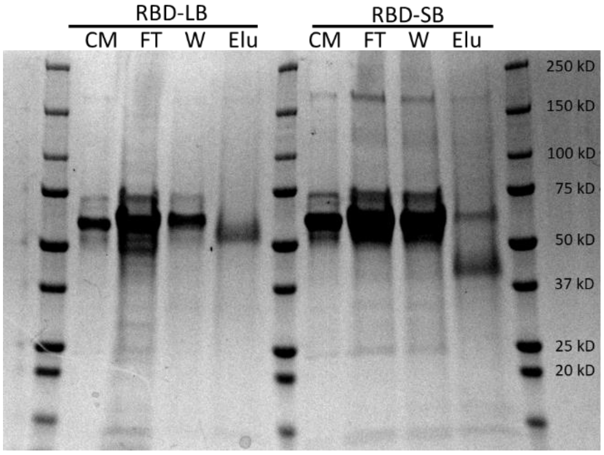

Supplementary Figure 30. SDS-PAGE analysis of RBD-SB and RBD-LB purification. The mammalian cell-expressed RBD-LB (58274 Da) and RBD-SB (42091 Da) proteins were purified by using Strep-Tactin chromatography. CM: culture media; FT/W/Elu: Strep-Tactin chromatography flow through, wash, and elution samples. The most intense band in the culture media is albumin (58 kDa). Gel image depicts an image from n = 1 independent experiments.

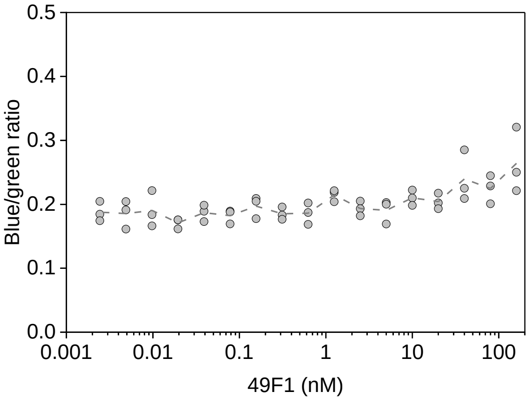

Supplementary Figure 31. **Ratiometric sensor response for anti-SARS-CoV-2-spike antibody 49F1.** Assays were performed using 1 nM RBD-LB and 1 nM RBD-SB in PBS buffer (pH 7.4, 0.1% (w/v) BSA) spiked with 0.5 pM calibrator luciferase. Sensor proteins were incubated with antibody 49F1 for 1 hour, followed by addition of NanoGlo substrate at 1000-fold final dilution. Antibody 49F1 binds to the region of spike protein outside the RBD, and therefore no obvious response was observed when using the split NanoLuc-functionalized RBD proteins to detect 49F1. Individual data points are represented as circles, and dashed line connects mean values (technical replicates, with n=3 independent preparations of the analyte). Source data are provided as a Source Data file.

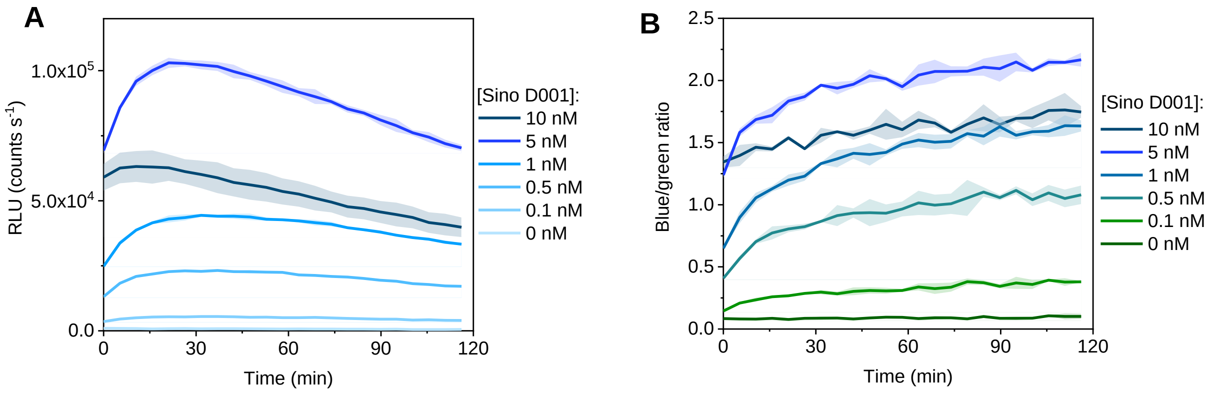

Supplementary Figure 32. Kinetics of one-step intensiometric and ratiometric assays for anti-SARS-CoV-2-spike antibody Sino D001. (**A**) Intensiometric assays using 0.5 nM RBD-LB and 0.5 nM RBD-SB in PBS buffer (pH7.4, 0.1% (w/v) BSA) with NanoGlo substrate at 1000-fold final dilution. (**B**) Ratiometric assays with addition of 0.3 pM calibrator luciferase. All components (sensor, calibrator, analyte and NanoGlo substrate) were added simultaneously at *t*=0. Data are represented as mean ± SD (technical replicates, with n=3 independent preparations of the analyte). Source data are provided as a Source Data file.

```
M  G  W  S  H  P  Q  F  E  K  G  G  S  M  T  F  K  L  I  I
ATGGGCTGGAGCCATCCGCAGTTTGAAAAAGGTGGTAGCATGACATTTAAACTGATTATC
N  G  K  T  L  K  G  E  I  T  I  E  A  V  D  A  *  E  A  E
AACGGCAAAACTTTAAAGGGAGAGATCACAATAGAAGCGGTGGATGCTTAGGAGGCGGAG
K  I  F  K  Q  Y  A  N  D  Y  G  I  D  G  E  W  T  Y  D  D
AAGATTTTTAAGCAGTATGCAAATGATTATGGAATTGATGGTGAATGGACTTATGACGAC
A  T  K  T  F  T  V  T  E  E  F  T  G  G  S  G  G  S  G  G
GCAACTAAAACTTTCACGGTAACAGAAGAATTTACAGGAGGTTCGGGTGGGTCGGGAGGT
S  G  G  S  G  G  S  G  G  S  G  E  F  A  E  A  A  A  K  E
TCTGGCGGCTCTGGAGGAAGTGGTGGTAGCGGTGAATTCGCCGAAGCAGCCGCTAAAGAA
A  A  A  K  E  A  A  A  K  E  A  A  A  K  E  A  A  A  K  E
GCCGCAGCAAAGGAAGCCGCGGCCAAGGAGGCAGCCGCAAAAGAGGCCGCGGCGAAGGAA
A  A  A  K  A  E  F  G  G  S  G  G  S  G  G  S  G  G  S  G
GCAGCAGCCAAGGCAGAATTCGGGGGTAGCGGCGGCTCGGGGGGTAGTGGTGGAAGCGGG
G  S  G  G  S  G  G  T  V  F  T  L  E  D  F  V  G  D  W  E
GGTTCAGGCGGTTCTGGGGGTACCGTCTTCACACTCGAAGATTTCGTTGGGGACTGGGAA
Q  T  A  A  Y  N  L  D  Q  V  L  E  Q  G  G  V  S  S  L  L
CAGACAGCCGCCTACAACCTGGACCAAGTCCTTGAACAGGGAGGTGTGTCCAGTTTGCTG
Q  N  L  A  V  S  V  T  P  I  Q  R  I  V  R  S  G  E  N  A
CAGAATCTCGCCGTGTCCGTAACTCCGATCCAAAGGATTGTCCGGAGCGGTGAAAATGCC
L  K  I  D  I  H  V  I  I  P  Y  E  G  L  S  A  D  Q  M  A
CTGAAGATCGACATCCATGTCATCATCCCGTATGAAGGTCTGAGCGCCGACCAAATGGCC
Q  I  E  E  V  F  K  V  V  Y  P  V  D  D  H  H  F  K  V  I
CAGATCGAAGAGGTGTTTAAGGTGGTGTACCCTGTGGATGATCATCACTTTAAGGTGATC
L  P  Y  G  T  L  V  I  D  G  V  T  P  N  M  L  N  Y  F  G
CTGCCCTATGGCACACTGGTAATCGACGGGGTTACGCCGAACATGCTGAACTATTTCGGA
R  P  Y  E  G  I  A  V  F  D  G  K  K  I  T  V  T  G  T  L
CGGCCGTATGAAGGCATCGCCGTGTTCGACGGCAAAAAGATCACTGTAACAGGGACCCTG
W  N  G  N  K  I  I  D  E  R  L  I  T  P  D  G  S  M  L  F
TGGAACGGCAACAAAATTATCGACGAGCGCCTGATCACCCCCGACGGCTCCATGCTGTTC
R  V  T  I  N  S  G  G  S  H  H  H  H  H  H  *
CGAGTAACCATCAACAGCGGAGGTTCCCACCACCACCATCACCACTAA
```

Supplementary Figure 33. DNA and amino acid sequence of Gx-LB. Strep-tag (gray), protein G (red), amber stop codon (yellow), LB (cyan), His-tag (dark red).

```
M  G  W  S  H  P  Q  F  E  K  G  G  S  M  T  F  K  L  I  I
ATGGGCTGGAGCCATCCGCAGTTTGAAAAAGGTGGTAGCATGACATTTAAACTGATTATC
N  G  K  T  L  K  G  E  I  T  I  E  A  V  D  A  *  E  A  E
AACGGCAAAACTTTAAAGGGAGAGATCACAATAGAAGCGGTGGATGCTTAGGAGGCGGAG
K  I  F  K  Q  Y  A  N  D  Y  G  I  D  G  E  W  T  Y  D  D
AAGATTTTTAAGCAGTATGCAAATGATTATGGAATTGATGGTGAATGGACTTATGACGAC
A  T  K  T  F  T  V  T  E  E  F  T  G  G  S  G  G  S  G  G
GCAACTAAAACTTTCACGGTAACAGAAGAATTTACAGGAGGTTCGGGTGGGTCGGGAGGT
S  G  G  S  G  G  S  G  G  S  G  E  F  A  E  A  A  A  K  E
TCTGGCGGCTCTGGAGGAAGTGGTGGTAGCGGTGAATTCGCCGAAGCAGCCGCTAAAGAA
A  A  A  K  E  A  A  A  K  E  A  A  A  K  E  A  A  A  K  E
GCCGCAGCAAAGGAAGCCGCGGCCAAGGAGGCAGCCGCAAAAGAGGCCGCGGCGAAGGAA
A  A  A  K  A  E  F  G  G  S  G  G  S  G  G  S  G  G  S  G
GCAGCAGCCAAGGCAGAATTCGGGGGTAGCGGCGGCTCGGGGGGTAGTGGTGGAAGCGGG
G  S  G  G  S  G  G  T  V  T  G  Y  R  L  F  E  K  E  S  G
GGTTCAGGCGGTTCTgggGGTACCGTTACCGGCTATCGTCTGTTTGAAAAAGAGAGCGGC
G  S  H  H  H  H  H  H  *
GGTTCACATCATCATCACCACCATTAA
```

Supplementary Figure 34. DNA and amino acid sequence of Gx-SB. Strep-tag (gray), protein G domain (red), amber stop codon (yellow), SB (blue), His-tag (dark red).

Gx-SB variants (SB only)

```
SB2 (Kd = 0.28 µM):   V  T  G  Y  R  L  F  E  K  I  L
                      GTTACCGGCTATCGTCTGTTTGAAAAAATTCTC
SB3 (Kd = 190 µM):    V  T  G  Y  R  L  F  E  E  I  L
                      GTTACCGGCTATCGTCTGTTTGAAGAAATTCTC
```

Supplementary Figure 35. DNA and amino acid sequences of SB in Gx-SB variants.

```
M  G  S  S  H  H  H  H  H  H  S  S  G  L  V  P  R  G  S  H
ATGGGCAGCAGCCATCATCATCATCATCACAGCAGCGGCCTGGTGCCGCGCGGCAGCCAT
M  S  D  S  E  V  N  Q  E  A  K  P  E  V  K  P  E  V  K  P
ATGTCGGACTCAGAAGTCAATCAAGAAGCTAAGCCAGAGGTCAAGCCAGAAGTCAAGCCT
E  T  H  I  N  L  K  V  S  D  G  S  S  E  I  F  F  K  I  K
GAGACTCACATCAATTTAAAGGTGTCCGATGGATCTTCAGAGATCTTCTTCAAGATCAAA
K  T  T  P  L  R  R  L  M  E  A  F  A  K  R  Q  G  K  E  M
AAGACCACTCCTTTAAGAAGGCTGATGGAAGCGTTCGCTAAAAGACAGGGTAAGGAAATG
D  S  L  R  F  L  Y  D  G  I  R  I  Q  A  D  Q  T  P  E  D
GACTCCTTAAGATTCTTGTACGACGGTATTAGAATTCAAGCTGATCAGACCCCTGAAGAT
L  D  M  E  D  N  D  I  I  E  A  H  R  E  Q  I  G  G  V  R
TTGGACATGGAGGATAACGATATTATTGAGGCTCACAGAGAACAGATTGGTGGTGTGCGT
S  S  S  R  T  P  S  D  K  P  V  A  H  V  V  A  N  P  Q  A
AGCAGCAGCCGTACCCCGAGCGATAAACCGGTGGCGCATGTGGTGGCGAATCCGCAGGCG
E  G  Q  L  Q  W  L  N  R  R  A  N  A  L  L  A  N  G  V  E
GAAGGCCAGCTGCAGTGGCTGAACCGTCGTGCGAATGCGCTGCTGGCCAACGGCGTGGAA
L  R  D  N  Q  L  V  V  P  S  E  G  L  Y  L  I  Y  S  Q  V
CTGCGTGATAATCAGCTGGTTGTGCCGAGCGAAGGCCTGTATCTGATTTATAGCCAGGTG
L  F  K  G  Q  G  C  P  S  T  H  V  L  L  T  H  T  I  S  R
CTGTTTAAAGGCCAGGGCTGCCCGAGCACCCATGTGCTGCTGACCCATACCATTAGCCGT
I  A  V  S  Y  Q  T  K  V  N  L  L  S  A  I  K  S  P  C  Q
ATTGCGGTGAGCTATCAGACCAAAGTGAACCTGCTGTCTGCGATTAAAAGCCCGTGCCAG
R  E  T  P  E  G  A  E  A  K  P  W  Y  E  P  I  Y  L  G  G
CGTGAAACCCCGGAAGGCGCGGAAGCGAAACCGTGGTATGAACCGATTTATCTGGGCGGC
V  F  Q  L  E  K  G  D  R  L  S  A  E  I  N  R  P  D  Y  L
GTGTTTCAGCTGGAAAAAGGCGATCGTCTGAGCGCGGAAATTAACCGTCCGGATTATCTG
D  F  A  E  S  G  Q  V  Y  F  G  I  I  A  L  G  S  G  G  S
GATTTTGCGGAAAGCGGCCAGGTGTATTTTGGCATTATTGCGCTGGGATCCGGGGGGAGC
G  G  S  G  G  S  G  G  S  G  G  S  G  G  S  G  G  S  G  G
GGTGGTAGCGGTGGGTCCGGGGGAAGTGGTGGCAGTGGTGGGTCCGGAGGGAGTGGCGGG
S  G  G  S  G  G  S  G  S  G  G  S  G  G  S  G  G  S  G  G
TCGGGAGGAAGCGGGGGCTCAGGATCCGGGGGCAGTGGCGGCTCAGGAGGTTCTGGAGGC
S  G  G  S  G  G  S  E  A  A  A  K  E  A  A  A  K  E  A  A
TCAGGCGGAAGCGGGGGTAGCGAAGCAGCCGCAAAAGAAGCAGCCGCCAAGGAAGCAGCA
A  K  E  A  A  A  K  E  A  A  A  K  E  A  A  A  K  G  G  S
GCAAAGGAGGCAGCTGCAAAGGAGGCGGCGGCGAAAGAGGCGGCAGCCAAAGGAGGATCT
G  G  S  G  G  S  G  G  S  G  G  S  G  G  S  G  T  V  T  G
GGTGGCTCAGGTGGTAGTGGAGGATCAGGAGGTAGTGGCGGATCAGGTACCGTTACCGGC
Y  R  L  F  E  K  E  S  G  G  S  G  G  S  W  S  H  P  Q  F
TATCGTCTGTTTGAAAAAGAGAGCGGTGGTTCAGGGGGCTCGTGGTCACATCCGCAATTT
E  K  *
GAAAAGTAA
```

Supplementary Figure 36. DNA and amino acid sequence of TNFα-SB. His-tag (dark red), SUMO-tag (yellow), TNFα (pink), SB (cyan), Strep-tag (gray).

```
M  G  I  L  P  S  P  G  M  P  A  L  L  S  L  V  S  L  L  S
ATGGGTATCCTTCCCAGCCCTGGGATGCCTGCGCTGCTCTCCCTCGTGAGCCTTCTCTCC
V  L  L  M  G  C  V  A  E  T  G  M  F  V  F  L  V  L  L  P
GTGCTGCTGATGGGTTGCGTAGCTGAAACCGGTATGTTCGTGTTTCTGGTGCTGCTGCCT
L  V  S  S  Q  R  V  Q  P  T  E  S  I  V  R  F  P  N  I  T
CTGGTGTCCAGCCAGCGGGTGCAGCCCACCGAATCCATCGTGCGGTTCCCCAATATCACC
N  L  C  P  F  G  E  V  F  N  A  T  R  F  A  S  V  Y  A  W
AATCTGTGCCCCTTCGGCGAGGTGTTCAATGCCACCAGATTCGCCTCTGTGTACGCCTGG
N  R  K  R  I  S  N  C  V  A  D  Y  S  V  L  Y  N  S  A  S
AACCGGAAGCGGATCAGCAATTGCGTGGCCGACTACTCCGTGCTGTACAACTCCGCCAGC
F  S  T  F  K  C  Y  G  V  S  P  T  K  L  N  D  L  C  F  T
TTCAGCACCTTCAAGTGCTACGGCGTGTCCCCTACCAAGCTGAACGACCTGTGCTTCACA
N  V  Y  A  D  S  F  V  I  R  G  D  E  V  R  Q  I  A  P  G
AACGTGTACGCCGACAGCTTCGTGATCCGGGGAGATGAAGTGCGGCAGATTGCCCCTGGA
Q  T  G  K  I  A  D  Y  N  Y  K  L  P  D  D  F  T  G  C  V
CAGACAGGCAAGATCGCCGACTACAACTACAAGCTGCCCGACGACTTCACCGGCTGTGTG
I  A  W  N  S  N  N  L  D  S  K  V  G  G  N  Y  N  Y  L  Y
ATTGCCTGGAACAGCAACAACCTGGACTCCAAAGTCGGCGGCAACTACAATTACCTGTAC
R  L  F  R  K  S  N  L  K  P  F  E  R  D  I  S  T  E  I  Y
CGGCTGTTCCGGAAGTCCAATCTGAAGCCCTTCGAGCGGGACATCTCCACCGAGATCTAT
Q  A  G  S  T  P  C  N  G  V  E  G  F  N  C  Y  F  P  L  Q
CAGGCCGGCAGCACCCCTTGTAACGGCGTGGAAGGCTTCAACTGCTACTTCCCACTGCAG
S  Y  G  F  Q  P  T  N  G  V  G  Y  Q  P  Y  R  V  V  V  L
TCCTACGGCTTTCAGCCCACAAATGGCGTGGGCTATCAGCCCTACAGAGTGGTGGTGCTG
S  F  E  L  L  H  A  P  A  T  V  C  G  P  K  K  S  T  N  L
AGCTTCGAACTGCTGCATGCCCCTGCCACAGTGTGCGGCCCTAAGAAAAGCACCAATCTC
V  K  N  K  C  V  N  F  F  T  G  G  S  G  G  S  G  G  S  G
GTGAAGAACAAATGCGTGAACTTCTTTACAGGAGGTTCGGGTGGGTCGGGAGGTTCTGGC
G  S  G  G  S  G  G  S  G  E  F  A  E  A  A  A  K  E  A  A
GGCTCTGGAGGAAGTGGTGGTAGCGGTGAATTCGCCGAAGCAGCCGCTAAAGAAGCCGCA
A  K  E  A  A  A  K  E  A  A  A  K  E  A  A  A  K  E  A  A
GCAAAGGAAGCCGCGGCCAAGGAGGCAGCCGCAAAAGAGGCCGCGGCGAAGGAAGCAGCA
A  K  A  E  F  G  G  S  G  G  S  G  G  S  G  G  S  G  G  S
GCCAAGGCAGAATTCGGGGGTAGCGGCGGCTCGGGGGGTAGTGGTGGAAGCGGGGGTTCA
G  G  S  G  G  S  V  F  T  L  E  D  F  V  G  D  W  E  Q  T
GGCGGTTCTGGGGGTAGCGTCTTCACACTCGAAGATTTCGTTGGGGACTGGGAACAGACA
A  A  Y  N  L  D  Q  V  L  E  Q  G  G  V  S  S  L  L  Q  N
GCCGCCTACAACCTGGACCAAGTCCTTGAACAGGGAGGTGTGTCCAGTTTGCTGCAGAAT
L  A  V  S  V  T  P  I  Q  R  I  V  R  S  G  E  N  A  L  K
CTCGCCGTGTCCGTAACTCCGATCCAAAGGATTGTCCGGAGCGGTGAAAATGCCCTGAAG
I  D  I  H  V  I  I  P  Y  E  G  L  S  A  D  Q  M  A  Q  I
ATCGACATCCATGTCATCATCCCGTATGAAGGTCTGAGCGCCGACCAAATGGCCCAGATC
E  E  V  F  K  V  V  Y  P  V  D  D  H  H  F  K  V  I  L  P
GAAGAGGTGTTTAAGGTGGTGTACCCTGTGGATGATCATCACTTTAAGGTGATCCTGCCC
Y  G  T  L  V  I  D  G  V  T  P  N  M  L  N  Y  F  G  R  P
TATGGCACACTGGTAATCGACGGGGTTACGCCGAACATGCTGAACTATTTCGGACGGCCG
Y  E  G  I  A  V  F  D  G  K  K  I  T  V  T  G  T  L  W  N
TATGAAGGCATCGCCGTGTTCGACGGCAAAAAGATCACTGTAACAGGGACCCTGTGGAAC
G  N  K  I  I  D  E  R  L  I  T  P  D  G  S  M  L  F  R  V
GGCAACAAAATTATCGACGAGCGCCTGATCACCCCCGACGGCTCCATGCTGTTCCGAGTA
T  I  N  S  G  G  S  G  T  L  E  V  L  F  Q  G  P  G  G  S
ACCATCAACAGCGGAGGTTCCGGTACCCTTGAGGTGCTGTTTCAGGGACCAGGAGGTAGT
G  S  A  W  S  H  P  Q  F  E  K  G  G  G  S  G  G  G  S  G
GGATCTGCTTGGAGCCATCCACAGTTCGAAAAAGGTGGAGGTTCTGGCGGTGGATCAGGT
G  S  A  W  S  H  P  Q  F  E  K  *
GGAAGTGCATGGTCTCACCCTCAGTTTGAGAAATAA
```

Supplementary Figure 37. DNA and amino acid sequence of RBD-LB. Chicken RPTPσ (receptor protein tyrosine phosphatase sigma) signal sequence (green), native N-terminal signal peptide of the spike protein (yellow), RBD (pink), LB (cyan), Strep-tag (gray).

```
M G I L P S P G M P A L L S L V S L L S
ATGGGTATCCTTCCCAGCCCTGGGATGCCTGCGCTGCTCTCCCTCGTGAGCCTTCTCTCC
V L L M G C V A E T G M F V F L V L L P
GTGCTGCTGATGGGTTGCGTAGCTGAAACCGGTATGTTCGTGTTTCTGGTGCTGCTGCCT
L V S S Q R V Q P T E S I V R F P N I T
CTGGTGTCCAGCCAGCGGGTGCAGCCCACCGAATCCATCGTGCGGTTCCCCAATATCACC
N L C P F G E V F N A T R F A S V Y A W
AATCTGTGCCCCTTCGGCGAGGTGTTCAATGCCACCAGATTCGCCTCTGTGTACGCCTGG
N R K R I S N C V A D Y S V L Y N S A S
AACCGGAAGCGGATCAGCAATTGCGTGGCCGACTACTCCGTGCTGTACAACTCCGCCAGC
F S T F K C Y G V S P T K L N D L C F T
TTCAGCACCTTCAAGTGCTACGGCGTGTCCCCTACCAAGCTGAACGACCTGTGCTTCACA
N V Y A D S F V I R G D E V R Q I A P G
AACGTGTACGCCGACAGCTTCGTGATCCGGGGAGATGAAGTGCGGCAGATTGCCCCTGGA
Q T G K I A D Y N Y K L P D D F T G C V
CAGACAGGCAAGATCGCCGACTACAACTACAAGCTGCCCGACGACTTCACCGGCTGTGTG
I A W N S N N L D S K V G G N Y N Y L Y
ATTGCCTGGAACAGCAACAACCTGGACTCCAAAGTCGGCGGCAACTACAATTACCTGTAC
R L F R K S N L K P F E R D I S T E I Y
CGGCTGTTCCGGAAGTCCAATCTGAAGCCCTTCGAGCGGGACATCTCCACCGAGATCTAT
Q A G S T P C N G V E G F N C Y F P L Q
CAGGCCGGCAGCACCCCTTGTAACGGCGTGGAAGGCTTCAACTGCTACTTCCCACTGCAG
S Y G F Q P T N G V G Y Q P Y R V V V L
TCCTACGGCTTTCAGCCCACAAATGGCGTGGGCTATCAGCCCTACAGAGTGGTGGTGCTG
S F E L L H A P A T V C G P K K S T N L
AGCTTCGAACTGCTGCATGCCCCTGCCACAGTGTGCGGCCCTAAGAAAAGCACCAATCTC
V K N K C V N F F T G G S G G S G G S G
GTGAAGAACAAATGCGTGAACTTCTTTACAGGAGGTTCGGGTGGGTCGGGAGGTTCTGGC
G S G G S G G S G E F A E A A A K E A A
GGCTCTGGAGGAAGTGGTGGTAGCGGTGAATTCGCCGAAGCAGCCGCTAAAGAAGCCGCA
A K E A A A K E A A A K E A A A K E A A
GCAAAGGAAGCCGCGGCCAAGGAGGCAGCCGCAAAAGAGGCCGCGGCGAAGGAAGCAGCA
A K A E F G G S G G S G G S G G S G G S
GCCAAGGCAGAATTCGGGGGTAGCGGCGGCTCGGGGGGTAGTGGTGGAAGCGGGGGTTCA
G G S G G S V T G Y R L F E K E S S G G
GGCGGTTCTGGGGGTAGCGTTACCGGCTATCGTCTGTTTGAAAAAGAGAGCAGCGGAGGT
S G T L E V L F Q G P G G S G S A W S H
TCCGGTACCCTTGAGGTGCTGTTTCAGGGACCAGGAGGTAGTGGATCTGCTTGGAGCCAT
P Q F E K G G G S G G G S G G S A W S H
CCACAGTTCGAAAAAGGTGGAGGTTCTGGCGGTGGATCAGGTGGAAGTGCATGGTCTCAC
P Q F E K *
CCTCAGTTTGAGAAATAA
```

Supplementary Figure 38. DNA and amino acid sequence of RBD-SB. Chicken RPTPσ (receptor protein tyrosine phosphatase sigma) signal sequence (green), native N-terminal signal peptide of the spike protein (yellow), RBD (pink), SB (cyan), Strep-tag (gray).

Supplementary tables

Supplementary Table 1. Sequences of primers used.

Primer name:	Short description	Primer sequence: (5' → 3')
SB2-Fw	Mutagenesis of SB (K_d = 2.5 µM) to SB2 (K_d = 0.28 µM), see Supplementary Fig. 9b and 35.	CGTCTGTTTGAAAAAATTCTCGGCGGTTCACATC
SB2-Rv	Mutagenesis of SB (K_d = 2.5 µM) to SB2 (K_d = 0.28 µM), see Supplementary Fig. 9b and 35.	GATGTGAACCGCCGAGAATTTTTTCAAACAGACG
SB3-Fw	Mutagenesis of SB (K_d = 2.5 µM) to SB3 (K_d = 190 µM), see Supplementary Fig. 9b and 35.	ATCGTCTGTTTGAAGAAATTCTCGGCGGTTCACATC
SB3-Rv	Mutagenesis of SB (K_d = 2.5 µM) to SB3 (K_d = 190 µM), see Supplementary Fig. 9b and 35.	GATGTGAACCGCCGAGAATTTCTTCAAACAGACGAT
LB-KpnI-remove-Fw	Used to generate DNA encoding RBD-LB, see Fig. 5e, Supplementary Fig. 30, 31, 32, 37 and 38.	GCGGTTCTGGGGGTAGCGTCTTCACACTCG
LB-KpnI-remove-Rv	Used to generate DNA encoding RBD-LB, see Fig. 5e, Supplementary Fig. 30, 31, 32, 37 and 38	CGAGTGTGAAGACGCTACCCCCAGAACCGC
SB-KpnI-remove-Fw	Used to generate DNA encoding RBD-SB, see Fig. 5e, Supplementary Fig. 30, 31, 32, 37 and 38	CGGTTCTGGGGGTAGCGTTACCGGCTATCG
SB-KpnI-remove-Rv	Used to generate DNA encoding RBD-SB, see Fig. 5e, Supplementary Fig. 30, 31, 32, 37 and 38	CGATAGCCGGTAACGCTACCCCCAGAACCG
SB-insert-KpnI-Rv	Used to generate DNA encoding RBD-SB, see Fig. 5e, Supplementary Fig. 30, 31, 32, 37 and 38	TATATAGGTACCTGAACCGCCGCTCTCTTTTTCAAAC
LB-KpnI-insert-Rv	Used to generate DNA encoding RBD-LB, see Fig. 5e, Supplementary Fig. 30, 31, 32, 37 and 38	TATATAGGTACCGGAACCTCCGCTGTTGATGGTTACTCG
RBD-overhang LB/SB-Rv	Used to generate DNA encoding RBD-LB and RBD-SB, see Fig. 5e, Supplementary Fig. 30, 31, 32, 37 and 38	CCGACCCACCCGAACCTCCTGTAAAGAAGTTCACGCATTTGTTC TTCACGAGATTGG
AgeI-RBD-Fw	Used to generate DNA encoding RBD-LB and RBD-SB, see Fig. 5e, Supplementary Fig. 30, 31, 32, 37 and 38	CCGACCGGTATGTTCGTGTTTCTGGTGCTGC
RBD overhang linker LB/SB-Fw	Used to generate DNA encoding RBD-LB and RBD-SB, see Fig. 5e, Supplementary Fig. 30, 31, 32, 37 and 38	ccaATCTCGTGAAGAACAAATGCGTGAACTTCTTTACAGGAGGTT CGGGTGGGTCGG

Supplementary Table 2. Overview of RAPPID assays for various protein targets. [a]

Target	Assay matrix [b]	Sensor concentrations	Calibrator concentrations	NanoGlo dilution (fold)	Incubation time (min) [c]	LOD (pM) [d]	Maximal ratio change (fold) [e]	Z'-factor [f]
cTnI (intensiometric)	PBS buffer	1 nM 19C7-LB, 1 nM 4C2-SB	-	400	30	4.2 (3.7, 5.0)	281	0.82
cTnI (ratiometric)	PBS buffer	1 nM 19C7-LB, 1 nM 4C2-SB	2 pM	400	30	19 (18, 21)	24	0.95
CRP	PBS buffer	1 nM C6-LB, 10 nM C135-SB	2 pM	400	30	2.9 (2.5, 3.4)	18	0.95
Anti-cetuximab	PBS buffer	1 nM CTX-LB, 1 nM CTX-SB	2 pM	1000	20	59 (50, 72)	6	0.96
Anti-adalimumab	PBS buffer	1 nM ADL-LB, 1 nM ADL-SB	2 pM	1000	20	68 (56, 87)	6	0.71
Anti-infliximab	PBS buffer	1 nM INFX-LB, 1 nM INFX-SB	2 pM	1000	20	81 (65, 109)	5	0.90
Adalimumab	10% plasma in PBS buffer	10 nM anti-ADL-LB 100 nM TNFα-SB	100 pM	1000	20	50 (46, 54)	11	0.97
Infliximab	10% plasma in PBS buffer	10 nM anti-INFX-LB 100 nM TNFα-SB	50 pM	1000	20	37 (33, 42)	6	0.96
SARS-CoV-2 Spike monomer	PBS buffer	1 nM 47D11-LB, 10 nM 49F1-SB	5 pM	400	60	1417 (1140, 1872)	2.5	0.82
SARS-CoV-2 Spike trimer	PBS buffer	1 nM 47D11-LB, 10 nM 47D11-SB	5 pM	400	60	1693 (1312, 2384)	3.5	0.80
SARS-CoV-2 Spike trimer	PBS buffer	1 nM 47D11-LB, 10 nM 49F1-SB	5 pM	400	60	1146 (958, 1426)	3.5	0.87
SARS-CoV-2 Spike trimer	PBS buffer	1 nM D001-LB, 10 nM D003-SB	10 pM	400	60	292 (267, 324)	5	0.97
SARS-CoV-2 Spike trimer	PBS buffer	1 nM D002-LB, 10 nM D004-SB	10 pM	400	60	236 (219, 256)	6	0.93

Anti-spike 47D11	PBS buffer	1 nM RBD-LB, 1 nM RBD-SB	0.2 pM	400	60	106 (87, 134)	10	0.87
Anti-spike Sino-D001	PBS buffer	1 nM RBD-LB, 1 nM RBD-SB	1 pM	400	60	16 (13, 22)	32	0.96
Anti-spike Sino-D003	PBS buffer	1 nM RBD-LB, 1 nM RBD-SB	0.5 pM	400	60	4.4 (3.4, 6.1)	36	0.93

[a] Most assays were performed without extensive optimization of the sensor, calibrator and NanoGlo concentrations as well as incubation time.
[b] Most assays were performed in PBS buffer (pH 7.4) containing 0.1% (w/v) BSA. The plasma content represents the final amount of plasma in assay mixtures.
[c] Preincubation of sensor proteins with analytes was used to allow sufficient complex formation. Shorter incubation may be applied according to the kinetics measurements shown in Figure 3D, Supplementary Figure 11, 16, 22, 25, 26 and 31.
[d] LOD was calculated from a single experiment performed in triplicate by linear regression of the response related to the analyte concentration for a limited range of concentrations. Values in the bracket represent 95% confidence interval of the LOD.
[e] Maximal ratio change was calculated by dividing the maximal emission ratio by the background emission ratio, i.e. the ratio at 0 nM analyte.
[f] Z'-factors were calculated according to Eq. 5 in Zhang et al.[7], defined as

$$Z' = 1 - 3\frac{\sigma_\mathrm{p} - \sigma_\mathrm{n}}{|\mu_\mathrm{p} - \mu_\mathrm{n}|}, \qquad \text{(S22)}$$

with μ_p and μ_n the mean of the peak value (maximum blue/green ratio or luminescent intensity, depending on the assay) and the negative value at 0 nM analyte, respectively, and σ_p and σ_n the corresponding standard deviations.

Supplementary Table 3. Quantification of CRP in 40 patient serum samples by using RAPPID and clinical method. Four patient samples with a concentration <4 mg/L were measured in both the clinical assay and RAPPID, and were omitted from analysis. Both methods could not distinguish below this cut-off value and this data was therefore not included in the method comparison.

RAPPID CRP concentration (mg/L)			Clinical method CRP concentration (mg/L)	
Triplo1	Triplo 2	Triplo 3	Duplo 1	Duplo 2
< 4	< 4	< 4	< 4	< 4
19	20	20	15	15
92	82	83	65	65
184	173	170	155	159
55	53	51	44	45
< 4	< 4	< 4	< 4	< 4
155	169	164	135	137
108	109	106	84	56
88	89	98	71	71
47	47	74	38	39
24	23	26	19	19
163	168	153	147	144
163	156	172	144	143
207	209	220	208	213
120	110	103	115	116
74	82	73	70	71
414	284	307	291	290
32	32	43	28	28
136	165	171	169	172
75	78	70	59	60
< 4	< 4	< 4	< 4	< 4
11	12	10	8	8
184	171	174	204	166
175	172	169	198	195
116	119	114	108	106
225	241	192	205	204
53	48	63	31	31
95	113	110	79	81
87	97	75	78	78
142	165	149	119	120
13	14	14	8	8
125	134	110	105	106
6	6	6	5	5
10	8	9	7	8
14	13	13	12	12
11	13	13	8	8
36	31	32	28	28
37	32	34	31	30
5	< 4	< 4	< 4	< 4
39	42	39	39	40

Supplementary references

1. Krishnamurthy, V. M., Semetey, V., Bracher, P. J., Shen, N. & Whitesides, G. M. Dependence of effective molarity on linker length for an intramolecular protein-ligand system. *J. Am. Chem. Soc.* **129**, 1312–1320 (2007).

2. Janssen, B. M. G. *et al.* Reversible blocking of antibodies using bivalent peptide–DNA conjugates allows protease-activatable targeting. *Chem. Sci.* **4**, 1442–1450 (2013).

3. Douglass, E. F., Miller, C. J., Sparer, G., Shapiro, H. & Spiegel, D. A. A comprehensive mathematical model for three-body binding equilibria. *J. Am. Chem. Soc.* **135**, 6092–6099 (2013).

4. Van Rosmalen, M., Krom, M. & Merkx, M. Tuning the flexibility of glycine-serine linkers to allow rational design of multidomain proteins. *Biochemistry* **56**, 6565–6574 (2017).

5. Zhou, H. X. Polymer models of protein stability, folding, and interactions. *Biochemistry* **43**, 2141–2154 (2004).

6. Schneider, C., Rasband, W. & Eliceiri, K. NIH image to ImageJ: 25 years of image analysis. *Nat. Methods* **9**, 671–675 (2012).

7. Zhang, J.-H., Chung, T. D. Y. & Oldenburg, K. R. A simple statistical parameter for use in evaluation and validation of high throughput screening assays. *J. Biomol. Screen.* **4**, 67–73 (1999).
