# Supplementary material for: A plug-and-play platform of ratiometric bioluminescent sensors for homogeneous immunoassays
Source: Nat Commun. 2021 Jul 28;12:4586. doi: 10.1038/s41467-021-24874-3 (PMC8319308; doi:10.1038/s41467-021-24874-3)
Supplement: Supplementary file 2 — Description of Additional Supplementary Files [file 41467_2021_24874_MOESM2_ESM.pdf]

Title: Supplementary Software

Description: This folder contains files for running simulations of sensor dose-response curves and estimating coefficients by using the thermodynamic model, elaborately described in the Supplementary Information. The code was written for use in MATLAB, and tested in MATLAB versions R2015a, R2019a, and R2020a. Normal personal computers were used, run with Windows 10 operating systems. No non-standard hardware is required. No installation is required, the zip file can be unzipped and the code files can be placed in a user-defined location.
