# Supplementary material for: A plug-and-play platform of ratiometric bioluminescent sensors for homogeneous immunoassays
Source: Nat Commun. 2021 Jul 28;12:4586. doi: 10.1038/s41467-021-24874-3 (PMC8319308; doi:10.1038/s41467-021-24874-3)
Supplement: Supplementary file 5 — Reporting Summary [file 41467_2021_24874_MOESM5_ESM.pdf]

nature research

Corresponding author(s): Maarten Merkx

Last updated by author(s): Jun 30, 2021

Reporting Summary

Nature Research wishes to improve the reproducibility of the work that we publish. This form provides structure for consistency and transparency in reporting. For further information on Nature Research policies, see our Editorial Policies and the Editorial Policy Checklist.

Statistics

For all statistical analyses, confirm that the following items are present in the figure legend, table legend, main text, or Methods section.

n/a	Confirmed	
☐	☒	The exact sample size (*n*) for each experimental group/condition, given as a discrete number and unit of measurement
☐	☒	A statement on whether measurements were taken from distinct samples or whether the same sample was measured repeatedly
☐	☒	The statistical test(s) used AND whether they are one- or two-sided<br>*Only common tests should be described solely by name; describe more complex techniques in the Methods section.*
☒	☐	A description of all covariates tested
☐	☒	A description of any assumptions or corrections, such as tests of normality and adjustment for multiple comparisons
☐	☒	A full description of the statistical parameters including central tendency (e.g. means) or other basic estimates (e.g. regression coefficient) AND variation (e.g. standard deviation) or associated estimates of uncertainty (e.g. confidence intervals)
☐	☒	For null hypothesis testing, the test statistic (e.g. *F*, *t*, *r*) with confidence intervals, effect sizes, degrees of freedom and *P* value noted<br>*Give P values as exact values whenever suitable.*
☒	☐	For Bayesian analysis, information on the choice of priors and Markov chain Monte Carlo settings
☒	☐	For hierarchical and complex designs, identification of the appropriate level for tests and full reporting of outcomes
☐	☒	Estimates of effect sizes (e.g. Cohen's *d*, Pearson's *r*), indicating how they were calculated

Our web collection on statistics for biologists contains articles on many of the points above.

Software and code

Policy information about availability of computer code

Data collection	Tecan SparkControl v2.1 (luminescence measurements)<br>SONY DSC-RX100 digital camera (luminescence recording)<br>GE Healthcare ImageQuant Capture v.1.0.2 (gel electrophoresis)<br>Waters MassLynx v4.1 (MS proteins)<br>NanoDrop ND-1000 v3.5.2 (concentration determination)<br>Promed UVL-30 UV light source (4×9 watt) (photoconjugation)
Data analysis	MATLAB R2015a, R2019a, and R2020a (thermodynamic model)<br>ImageJ 1.51j8 (image analysis)<br>Origin v2020 (plotting and data fitting)<br>Microsoft Excel for Office 365 MSO (calculation of limits of detection and plotting in supplementary information)<br>MagTran v1.03 (MS deconvolution)<br>MassLynx 4.1 (SCN862) software (proteins mass analysis)<br>Snapgene 5.2.4 (cloning design and sequence verification)

For manuscripts utilizing custom algorithms or software that are central to the research but not yet described in published literature, software must be made available to editors and reviewers. We strongly encourage code deposition in a community repository (e.g. GitHub). See the Nature Research guidelines for submitting code & software for further information.

Data

Policy information about availability of data

All manuscripts must include a data availability statement. This statement should provide the following information, where applicable:

- Accession codes, unique identifiers, or web links for publicly available datasets
- A list of figures that have associated raw data
- A description of any restrictions on data availability

All raw data from luminescent assays that have been used to generate the figures in the manuscript are available in the Source Data files. There are no restrictions on data availability.

Field-specific reporting

Please select the one below that is the best fit for your research. If you are not sure, read the appropriate sections before making your selection.

☒ Life sciences ☐ Behavioural & social sciences ☐ Ecological, evolutionary & environmental sciences

For a reference copy of the document with all sections, see nature.com/documents/nr-reporting-summary-flat.pdf

Life sciences study design

All studies must disclose on these points even when the disclosure is negative.

Sample size	No statistical methods were used to predetermine sample size. We performed all luminescence measurements in triplicate (Fig. 2d, 2c, 2d, 2e, 2f, 2g, 2h, 3b, 3c, 4b, 4d, 4e, 4f, 5b, 5c and 5e) and found the sample size adequate as the data was reproducible, as indicated. The luminescence measurements in Fig. 3d were performed in quadruplicate. We measured 40 CRP patient samples either in triplicate or in duplicate for our RAPPID assay or the clinical assay, respectively. This sample size was chosen based on standard protocols used and approved in clinical routine to enable a method comparison experiment to determine if the performance of our assay and the clinical assay are statistically identical.
Data exclusions	Four patient samples with a concentration <4 mg/L were measured in both the clinical assay and RAPPID, and were omitted from further analysis. Both methods could not distinguish below this cut-off value and this data was therefore not included in the method comparison.
Replication	All attempts at reproducing the results in this study were successful. The luminescence data represent a single experiment performed in triplicate or quadruplicate, wherein the analytes were independently prepared by dilution.
Randomization	This is not relevant for this observational study. We do not include treatment and this study will therefore not be affected by the relevant bias.
Blinding	Experiments with CRP patient samples were performed in blinded mode, with all samples de-identified before measurement. For the other luminescence measurements blinding was not relevant as we spiked buffer or pooled plasma with known concentrations of analyte.

Reporting for specific materials, systems and methods

We require information from authors about some types of materials, experimental systems and methods used in many studies. Here, indicate whether each material, system or method listed is relevant to your study. If you are not sure if a list item applies to your research, read the appropriate section before selecting a response.

Materials & experimental systems

n/a	Involved in the study	
☐	☒	Antibodies
☐	☒	Eukaryotic cell lines
☒	☐	Palaeontology and archaeology
☒	☐	Animals and other organisms
☐	☒	Human research participants
☒	☐	Clinical data
☒	☐	Dual use research of concern

Methods

n/a	Involved in the study	
☒	☐	ChIP-seq
☒	☐	Flow cytometry
☒	☐	MRI-based neuroimaging

Antibodies

Antibodies used	- Monoclonal mouse anti-cardiac troponin I; Supplier: Hytest; Catalogue: 4T21; MAb: 19C7; Lot: 18/04-T21-C7.<br>- Monoclonal mouse anti-cardiac troponin I; Supplier: Hytest; Catalogue: 4T21; MAb: 4C2; Lot: 18/04-T21-C2.<br>- Monoclonal mouse anti-human C-reactive protein (high sensitivity CRP); Supplier: Hytest; Catalogue: 4C28 / 4C28cc; MAb: C6cc; Lot:16/02-C28cc-C6cc.<br>- Monoclonal mouse anti-human C-reactive protein (high sensitivity CRP); Supplier: Hytest; Catalogue: 4C28 / 4C28cc; MAb: CRP135;

Lot: 17/02-C28-CRP135.
- Human Anti Infliximab; Supplier: BioRad; Catalogue: HCA213; Clone: AbD17841_hIgG1. Batch No: 1612.
- Human Anti Infliximab; Supplier: BioRad; Catalogue: HCA233; Clone: AbD20436_hIgG1. Lot: 1807.
- Human Anti Cetuximab; Supplier: BioRad; Catalogue: HCA221; Clone: AbD19830_hIgG1. Batch No: 1607.
- Human Anti Adalimumab (Drug/Target Complex); Supplier: BioRAd; Catalogue: HCA207; Clone: AbD18754_hIgG1. Lot: 0912R.
- Anti-TNF-α (Adalimumab), humanized Antibody; Supplier: Gentaur; Catalogue: A1048-100; Identity: 11892. Lot. 5D05 A10480.
- Cetuximab; Supplier: Merck Serono. Name of medicine: Erbitux 5 mg/mL solution for infusion. MA (EU) Number: EU/1/04/281/005.
- Infliximab; Supplier: Hospira. Name of medicine: Inflectra 100 mg.
- Primary antibodies 47D11 and 49F1 were produced in house as described in reference 44: Wang, C. et al. A human monoclonal antibody blocking SARS-CoV-2 infection. Nat. Commun. 11, 2251 (2020).
- SARS-CoV/SARS-CoV-2 Spike antibody,Chimeric MAb; Supplier: Sino Biological; Catalogue: 40150-D001; Clone ID: D001.
- SARS-CoV/SARS-CoV-2 Spike antibody,Chimeric MAb; Supplier: Sino Biological; Catalogue: 40150-D002; Clone ID: D002.Lot: MA14AP0603.
- SARS-CoV/SARS-CoV-2 Spike antibody,Chimeric MAb; Supplier: Sino Biological; Catalogue: 40150-D003; Clone ID: D003. Lot: HA4AP2304.
- SARS-CoV/SARS-CoV-2 Spike antibody,Chimeric MAb; Supplier: Sino Biological; Catalogue: 40150-D004; Clone ID: D004. Lot: MA14AP0203.

Validation

- Both anti-cTnI antibodies were validated by the manufacturer for immunoassays, immunoprecipitation, immunohistochemistry, immunoaffinity purification, and Western blotting.
- Both anti-CRP antibodies were validated by the manufacturer for Human C-reactive protein immunodetection in direct ELISA, high sensitivity sandwich immunoassay, competitive immunoassays, turbidimetric assays, immunoaffinity purification, and immunohistochemistry.
- Human anti-infliximab antibody HCA213 was validated by the manufacturer for direct ELISA, bridging ELISA, and for ADA assays. References: (1) Hernández-Breijo, B. et al. (2015) Quantification of the concentration of antibodies against Infliximab in human serum using a pure antibody as calibrator. 10th Congress of ECCO, Barcelona P020. (2) Hernández-Breijo, B. et al. (2016) Standardization of the homogeneous mobility shift assay protocol for evaluation of anti-infliximab antibodies. Application of the method to Crohn's disease patients treated with infliximab. Biochem Pharmacol. Sep 21. pii: S0006-2952(16)30302-1.
- Human anti-infliximab antibody HCA233 was validated by the manufacturer for direct ELISA and bridging ELISA in ADA assays. References: (1) Lee, M.W. et al. (2016) Comparison of infliximab drug measurement across three commercially available ELISA kits.
- Human anti-cetuximab antibody was validated by the manufacturer for direct ELISA and bridging ELISA in ADA assays. Reference: Gomez, D. et al. (2013) Cetuximab therapy in the treatment of metastatic colorectal cancer: the future frontier? Int J Surg. 11 (7): 507-13.
- Human anti-adalimumab antibody was validated by the manufacturer for indirect ELISA on adalimumab/TNFα complexes and for use as a detection antibody, when conjugated to HRP, for adalimumab bound to TNFα. References for Adalimumab antibody: Harth, S. et al. (2019) Generation by phage display and characterization of drug-target complex-specific antibodies for pharmacokinetic analysis of biotherapeutics.
MAbs. 11 (1): 178-190.
- Adalimumab: has been validated for neutralization experiments and ELISA by the manufacturer.
- Cetuximab: has been validated for therapeutic use by the manufacturer.
- Infliximab: has been validated for therapeutic use by the manufacturer.
- Primary antibodies 47D11 and 49F1 were validated by different assays including ELISA, IFA and FACS, as described in reference 52: Wang, C. et al. A human monoclonal antibody blocking SARS-CoV-2 infection. Nat. Commun. 11, 2251 (2020).
- SARS-CoV/SARS-CoV-2 Spike antibodies were validated by the manufacturer for ELISA. Citation: Tan X. et al. Rapid and quantitative detection of SARS-CoV-2 specific IgG for convalescent serum evaluation.Biosens Bioelectron. 169 (2020) 112572.

Eukaryotic cell lines

Policy information about cell lines

Cell line source(s)	Human Embryonic Kidney Cells, 293T, from ATCC. ATCC Number: CRL-3216. Lot Number: 70023985
Authentication	All cells were authenticated by ATCC, with a COI assay (interspecies), STR analysis (interspecies), sterility test and human pathogenic virus testing (PCR-based assay).
Mycoplasma contamination	We confirm that the cells were tested as mycoplasma negative.
Commonly misidentified lines (See ICLAC register)	No commonly misidentified cell lines were used.

Human research participants

Policy information about studies involving human research participants

Population characteristics	Experiments with 40 CRP patient samples were done in blinded mode. Therefore, the population characteristics are unknown.
Recruitment	Only samples were used in which CRP was ordered as part of standard clinical care and of patients who did not object against usage of their remnant blood for quality purposes. 40 CRP patient samples were chosen randomly and in blinded mode from the CRP blood samples that were drawn the day before the assays were performed in the Rijnstate Hospital, thereby preventing bias.
Ethics oversight	All patient samples were obtained under protocols and guidelines approved by the local ethical commitee of the Rijnstate Hospital (reference number: KCHL 2021-1790) and in accordance with the Declaration of Helsinki, and de-identified before use. All patients provided informed consent.

Note that full information on the approval of the study protocol must also be provided in the manuscript.
